# Genome-Wide Association Study Identifies Novel Candidate Variants Associated with Postoperative Nausea and Vomiting

**DOI:** 10.3390/cancers15194729

**Published:** 2023-09-26

**Authors:** Daisuke Nishizawa, Ryozo Morino, Rie Inoue, Seii Ohka, Shinya Kasai, Junko Hasegawa, Yuko Ebata, Kyoko Nakayama, Hiroyuki Sumikura, Masakazu Hayashida, Miyuki Yokota, Kazutaka Ikeda

**Affiliations:** 1 Addictive Substance Project, Tokyo Metropolitan Institute of Medical Science, Tokyo 156-8506, Japan; nishizawa-ds@igakuken.or.jp (D.N.);; 2 Division of Anesthesiology, Koujinkai Daiichi Hospital, Tokyo 125-0041, Japan; 3 Department of Anesthesiology and Pain Medicine, Juntendo University School of Medicine, Tokyo 113-8421, Japan; 4 Department of Anesthesiology, Cancer Institute Hospital, Tokyo 135-8550, Japan; 5 Department of Anesthesiology, East Hokkaido Hospital, Kushiro 085-0036, Japan

**Keywords:** postoperative nausea and vomiting, anesthesia, propofol, single-nucleotide polymorphisms, genome-wide association study

## Abstract

**Simple Summary:**

Postoperative nausea and vomiting (PONV) is experienced by approximately 30% of patients who undergo general anesthesia. However, many genetic factors involved in the vulnerability to PONV remain unidentified. The aim of our genome-wide association study (GWAS) was to comprehensively explore genetic variations associated with PONV. We identified several single-nucleotide polymorphisms (SNPs) that may possibly be associated with the frequency of nausea and vomiting, of which the most potent were the rs2776262, rs140703637, rs7212072, rs12444143, rs45574836, and rs1752136 SNPs. These results indicate that these SNPs in the *LOC100506403*, *CNTN5*, *SHISA6*, *RBFOX1*, *ATP8B3*, and *LOC105370198* gene regions could serve as markers that predict the vulnerability to PONV.

**Abstract:**

Considerable individual differences are widely observed in the incidence of postoperative nausea and vomiting (PONV). We conducted a genome-wide association study (GWAS) to identify potential candidate single-nucleotide polymorphisms (SNPs) that contribute to PONV by utilizing whole-genome genotyping arrays with more than 950,000 markers. The subjects were 806 patients who provided written informed consent and underwent elective surgery under general anesthesia with propofol or desflurane. The GWAS showed that two SNPs, rs2776262 and rs140703637, in the *LOC100506403* and *CNTN5* gene regions, respectively, were significantly associated with the frequency of nausea. In another GWAS conducted only on patients who received propofol, rs7212072 and rs12444143 SNPs in the *SHISA6* and *RBFOX1* gene regions, respectively, were significantly associated with the frequency of nausea as well as the rs2776262 SNP, and the rs45574836 and rs1752136 SNPs in the *ATP8B3* and *LOC105370198* gene regions, respectively, were significantly associated with vomiting. Among these SNPs, clinical and SNP data were available for the rs45574836 SNP in independent subjects who underwent laparoscopic gynecological surgery, and the association was replicated in these subjects. These results indicate that these SNPs could serve as markers that predict the vulnerability to PONV. Our findings may provide valuable information for achieving satisfactory prophylactic treatment for PONV.

## 1. Introduction

General anesthesia is commonly utilized in surgeries for the treatment of cancer and other diseases. Postoperative nausea and vomiting (PONV) is the most common adverse event following general anesthesia [1], with an estimated incidence of 30% in the general surgical population and as high as 80% in high-risk cohorts [1,2,3]. The development of PONV is associated with significantly lower postoperative quality of life (QOL) [1,4]. Unresolved PONV may result in prolonged post-anesthesia stays in the care unit or hospital that can significantly increase overall healthcare costs [3]. PONV is thought to be multifactorial, involving anesthetic, surgical, and individual risk factors [2,5,6,7]. Female gender, a history of PONV, non-smoking status, a history of motion sickness, and younger age are patient-specific predictors, and the use of volatile anesthetics, the duration of anesthesia, postoperative opioid use, and nitrous oxide have been reported to be anesthesia-related predictors. Cholecystectomy, gynecological surgery, and laparoscopic procedures have been shown to be surgical risk factors for PONV [8,9,10]. Among these, four major factors—female gender, a history of PONV and/or motion sickness, non-smoking status, and the use of postoperative opioids—were incorporated into a simplified risk score to predict PONV that was developed by Apfel et al. (1999) [2]. However, even patients at low PONV risk according to their assigned Apfel score may experience PONV, suggesting a genetic predisposition [11].

Previous genetic studies of candidate molecules that are related to nausea/vomiting mechanisms identified human genetic variants associated with PONV-related phenotypes in genes that encode serotonin receptor type 3, dopamine receptor type 2, μ-opioid receptor, neurokinin 1 receptor, serotonin transporter, adenosine triphosphate (ATP)-binding cassette subfamily B member 1 transporter, organic cation transporter (OCT), and cytochrome P450 2D6 isoform, among others [11,12,13,14]. To date, genetic polymorphisms within and around several genes, including *HTR3A* [15,16], *HTR3B* [16,17,18], *DRD2*/*ANKK1* [19,20], *OPRM1* [21,22], *TACR1* [23], *SLC6A4* [24], *ABCB1* [25,26,27], *OCT1* (*SLC22A1*) [28], and *CYP2D6* [29,30,31], have been found to be associated with PONV-related phenotypes. PONV-related single-nucleotide polymorphisms (SNPs) have also been comprehensively explored based on recent advances in high-density SNP arrays that can screen hundreds of thousands or millions of genetic markers throughout the human genome. For example, Janicki et al. (2011) found that one SNP in the *CHRM3* gene, rs2165870, which encodes muscarinic acetylcholine receptor 3, was associated with PONV in a genome-wide association study (GWAS) using pooled deoxyribonucleic acid (DNA) and a separate verification study [32]. Klenke et al. (2018) confirmed the association between the rs2165870 SNP and PONV [33] and identified another SNP, rs349358, in the gene that encodes potassium voltage-gated channel subfamily B member 2 (*KCNB2*), which was significantly associated with PONV [14]. Another GWAS was reported in female subjects who had a higher risk of PONV and underwent breast cancer surgery with standardized propofol anesthesia and antiemetics, in which six variants with a suggestive association with PONV (*p* < 1 × 10^−5^) were identified, and the association with the *DRD*2 variant rs1800497 (TaqIA) in previous studies [19,20] was replicated [34]. Although the aforementioned GWASs were all conducted in subjects of European origin, a GWAS in subjects of Asian origin was also recently reported [35]. Sugino et al. (2020) performed a GWAS using a DNA microarray that was optimized for genotyping in Japanese populations and identified 78 SNPs that were associated with the incidence of PONV in a limited sample of 24 female patients (*p* < 1 × 10^−4^). Among these, associations of the two candidate SNPs, the rs1333114 SNP of the *PTPRD* gene and the rs11232965 SNP of the *MIR4300HG* gene, were verified in independent samples [35]. However, GWASs with relatively large sample sizes have not been conducted in Asian populations to date, and many genetic factors that contribute to PONV remain unknown.

In the present study, we conducted a GWAS on subjects who were scheduled to undergo general anesthesia by total intravenous anesthesia (TIVA) with propofol or inhalational anesthesia with desflurane to identify potential genetic variants contributing to the vulnerability to PONV. Considering that the entire population was a mixture of subjects who underwent general anesthesia with propofol and desflurane, another GWAS was also performed only in subjects who underwent general anesthesia with propofol to explore the genetic factors associated with PONV.

## 2. Materials and Methods

### 2.1. Patients

#### 2.1.1. Patients Who Underwent Elective Surgery under General Anesthesia with Propofol or Desflurane

Enrolled in the study were 806 adult patients (20–93 years old, 432 males and 374 females) who were scheduled to undergo elective surgery for cancer under general anesthesia by TIVA with propofol or inhalational anesthesia with desflurane at The Cancer Institute Hospital of the Japanese Foundation for Cancer Research (JFCR; CIH samples). The exclusion criteria were the following: (1) patients to whom mild or more emetogenic antitumor agents were administered or were scheduled to be administered from 6 days before the start of the study to 48 h after surgery; (2) patients with symptomatic brain metastases; (3) patients who used the following antiemetic drugs within 48 h before and during surgery: 5-hydroxytryptamine 3 (5-HT_3_) receptor antagonists (granisetron, ondansetron, azasetron, etc.), phenothiazines (chlorpromazine, prochlorperazine, perphenazine, etc.), butyrophenone-based preparations (haloperidol, droperidol, etc.), benzamide preparations (sulpiride, tiapride, sultopride, etc.), dopamine receptor antagonists (metoclopramide, itopride, domperidone, etc.), antihistamines (hydroxyzine, dimenhydrinate, diphenhydramine), or NK1 receptor antagonists (apireptant); (4) patients who were mentally unable to communicate; (5) patients who were pregnant; (6) patients who were judged to be inappropriate for inclusion in the study by the investigator; and (7) patients of the Head and Neck Department and Gastroenterology Department who needed advanced management in the postoperative intensive care unit. The major reasons for applying these exclusion criteria were the possible influence of these factors on the incidence and severity of PONV and the collection of accurate data. The cancellation criteria were the following: (1) patients for whom blood collection was not possible and (2) patients whose informed consent was withdrawn. All of the individuals who were included in the study were of Japanese origin. Peripheral blood samples were collected from these subjects for gene analysis. Detailed demographic and clinical data of the subjects are provided in Table 1.

The study was conducted according to the guidelines of the Declaration of Helsinki and approved by the Institutional Review Board or Ethics Committee of The Cancer Institute Hospital and Tokyo Metropolitan Institute of Medical Science (Tokyo, Japan). Written informed consent was obtained from all of the patients.

#### 2.1.2. Patients Who Underwent Laparoscopic Gynecological Surgery

Enrolled in the study were 350 females (20–70 years old) who were classified as American Society of Anesthesiologists (ASA) Physical Status (ASA-PS) Class I or II and were scheduled to undergo laparoscopic gynecological surgery (LGS) under general anesthesia for benign gynecological diseases (e.g., uterine myoma and ovarian cysts) at Juntendo University Hospital between June 2017 and May 2019 (JUH samples). Excluded were patients who chronically received antipsychotic drugs, antiepileptic drugs, or opioid analgesics; patients with obstructive sleep apnea syndrome; and patients whose body mass index (BMI) was >30 kg/m^2^. Additionally, patients for whom surgery was converted from LGS to open abdominal surgery and patients who underwent re-operation for hemorrhage were excluded. The major reasons for applying these exclusion criteria were the possible influence of these factors on the incidence and severity of PONV and the collection of accurate data.

The study was conducted according to the guidelines of the Declaration of Helsinki and was approved by the Institutional Review Board or Ethics Committee of Juntendo University School of Medicine and Tokyo Metropolitan Institute of Medical Science (Tokyo, Japan). Written informed consent was obtained from all of the patients.

### 2.2. Patient Characteristics and Clinical Data

#### 2.2.1. Patient Characteristics and Clinical Data in Patients Who Underwent Elective Surgery under General Anesthesia with Propofol or Desflurane

In the CIH samples, we obtained data on patient characteristics (gender, age, height, weight, and BMI), history of smoking, frequency of alcohol drinking per week, history of motion sickness, history of PONV, surgery data, clinical data for the postoperative period (duration of anesthesia, duration of surgery, type of anesthesia, total dose of remifentanil, total dose of fentanyl, postoperative administration of narcotic drugs, and postoperative administration of opioids (including pentazocine)), the experience and frequency of postoperative pain, and PONV data (Table 1).

For PONV evaluation, the evaluator recorded the number of nausea and vomiting episodes that occurred in the postoperative period. Vomiting was defined as episodes of vomiting and/or retching that occurred once or more (the act of excreting the contents of the stomach) or dry vomiting (the act of trying to vomit without excreting the contents of the stomach). Vomiting that occurred as separate events was defined as the absence of vomiting for at least 1 min between two events. The investigator thoroughly explained the definition of vomiting to the subject.

The presence or absence of nausea, frequency of nausea, presence or absence of vomiting, and presence or absence of PONV (the presence or absence of the incidence of nausea and/or vomiting) were used as endpoints for the genetic association analysis in the present study. Despite possible correlations among the four major endpoint variables, GWASs were performed for all four of these phenotypes in case even slight differences in these endpoint values could be caused by some slightly or moderately different genetic variants. The frequency of vomiting was not analyzed because vomiting occurred only once in most of the subjects. The clinical data on the subjects are detailed in Table 1. Although several kinds of opioids were administered during surgery and the postoperative period, opioid narcotic administration was not standardized in the present study, because opioid administration during surgery and the postoperative period were treated as different variates in the clinical data analyses.

#### 2.2.2. Patient Characteristics and Clinical Data in Patients Who Underwent Laparoscopic Gynecological Surgery

For the JUH samples, patient characteristics, surgical protocols, and postoperative pain management are detailed in another report by Inoue et al. (in preparation). Briefly, anesthesia was induced with remifentanil at a rate of 0.5 μg/kg/min and the target-controlled infusion (TCI) of propofol at a target concentration of 3–5 μg/mL using a TCI pump (TE-371, Terumo, Tokyo, Japan). Dexamethasone (6.6 mg) and droperidol (1.25 mg) were administered intravenously (i.v.) to prevent PONV. Around the end of surgery, infusions of propofol and remifentanil were discontinued. Fentanyl (approximately 4 μg/kg) and acetaminophen (20 mg/kg, up to 1000 mg) were administered i.v. to achieve immediate postoperative pain relief. When patients complained of significant pain, fentanyl (50–100 μg) was given in increments. After adequate immediate postoperative pain relief was achieved, postoperative pain was managed with i.v. fentanyl patient-controlled analgesia (PCA) combined with droperidol (fentanyl (1000 μg in 20 mL) and droperidol (2.5 mg in 1 mL) diluted with normal saline (80 mL) to a total volume of 101 mL) that commenced using a CADD-Legacy PCA pump (Smiths Medical Japan, Tokyo, Japan). Additionally, acetaminophen (20 mg/kg, up to 1000 mg) was administered i.v. every 6 h until it became unnecessary during the first 24 h postoperative period. When the analgesia that was achieved with i.v. fentanyl PCA combined with repeated doses of acetaminophen was inadequate, i.v. flurbiprofen axetil (50 mg) or i.v. pentazocine (30 mg) were given as rescue analgesics. The presence or absence of PONV was assessed at the same time as pain intensities, every 3 h postoperatively or whenever patients complained of PONV. When required, i.v. metoclopramide (10 mg) or rectal domperidone (60 mg) were used to treat PONV. The number of patients who experienced PONV or other adverse effects of fentanyl within the 24 h postoperative period was noted. The characteristics of these clinical data are summarized in Supplementary Table S1.

### 2.3. Whole-Genome Genotyping and Quality Control

For the CIH samples, 10 mL of venous blood was sampled during anesthesia for the subsequent preparation of genomic DNA specimens. The DNA concentration was adjusted to 100 ng/L for whole-genome genotyping using a NanoDrop ND-1000 Spectrophotometer (NanoDrop Technologies, Wilmington, DE, USA). Whole-genome genotyping was performed using Infinium Assay II with an iScan system (Illumina, San Diego, CA, USA), according to the manufacturer’s instructions. Three kinds of BeadChips were used to genotype 806 samples: HumanOmniExpressExome-8 v. 1.2 (total markers: 964,193), HumanOmniExpressExome-8 v. 1.3 (total markers: 958,497), and HumanOmniExpressExome-8 v. 1.4 (total markers: 960,919). The BeadChips included a number of probes that were specific to copy number variation markers, but most of the BeadChips were for SNP markers on the human autosomes or sex chromosomes. Approximately 946,000 SNP markers were commonly included in all of the BeadChips.

For the JUH samples, 10 mL of venous blood was also sampled to prepare DNA specimens. According to the manufacturer’s recommendations, whole-genome genotyping was performed using Infinium Assay II with an iScan system (Illumina). Infinium Asian Screening Array-24 v. 1.0 BeadChips (one kind) were utilized to genotype 333 patient samples (total markers: 659,184). Numerous copy number variation markers were included in the BeadChips, but the majority of the BeadChips were for SNP markers on the human autosomes or sex chromosomes.

GenomeStudio v. 2.0.4 with the Genotyping v. 2.0.4 module (Illumina) was used to examine the data for samples with their entire genomes genotyped to assess the quality of the findings. Following data cleaning, quality control was performed as for the CIH samples. The patient samples retained a total of 651,086 SNP markers after this screening step. Among these SNPs, the genotype data for a potent SNP found in the GWAS, rs45574836 (exm1401859), were extracted and used for a further replication study.

In the CIH samples, for phenotypes of the frequency of nausea in all patients, the frequency of nausea in patients who received propofol, and the presence/absence of vomiting in patients who received propofol, log QQ *p*-value plots were subsequently drawn as a result of the GWAS for the 806 samples to check the pattern of the generated *p*-value distribution, in which the observed *p*-values against the values that were expected from the null hypothesis of a uniform distribution, calculated as −log10 (*p* value), were plotted for each model. All of the plots were mostly concordant with the expected line (y = x), especially over the range of 0 < −log10 (*p* value) < 2.5 for each model in the frequency of nausea in patients who received propofol and vomiting in patients who received propofol, and over the range of 0 < −log10 (*p* value) < 4.5 for each model in vomiting in patients who received propofol, indicating no apparent population stratification of the samples that were used in the study (Supplementary Figures S1–S3). The Functional Mapping and Annotation of Genome-Wide Association Studies (FUMA GWAS) v. 1.3.9 platform was used to visualize the QQ plots [36].

### 2.4. Statistical Analysis

In the GWAS for CIH samples, the presence/absence of nausea, frequency of nausea, presence/absence of vomiting, and presence/absence of PONV (the presence/absence of nausea or vomiting) were used as indices of the vulnerability or severity of PONV during the 48 h postoperative period. Before the analyses, quantitative values of the frequency of nausea, represented as integer numbers of the incidence of nausea during the 48 h postoperative period, were modified based on the procedure of variable transformation that was developed by Yeo and Johnson (2000), with the lambda value set as 0.5 for approximation to the normal distribution [37]. To explore the associations between SNPs and the incidence of PONV, Fisher’s exact test or the Cochran–Armitage trend test were conducted in analyses using both the group of all patients and that of patients who received propofol. This was carried out to compare genotype data between subjects with the presence of incidence and subjects with the absence of incidence. To explore associations between the SNPs and the frequency of nausea in analyses using both the group of all patients and that of patients who received propofol, linear regression analyses were conducted in which the variable-transformed frequency of nausea and genotype data for each SNP were incorporated as dependent and independent variables, respectively, with proper covariates. Trend or additive, dominant, and recessive genetic models were used for the analyses due to our previously insufficient knowledge about genetic factors that are associated with PONV. Male genotypes were not included in the analysis of X chromosome markers, whereas both male and female individuals were included in the association study for autosomal markers. PLINK v. 1.07 (https://zzz.bwh.harvard.edu/plink/index.shtml; accessed on 25 June 2023) [38], gPLINK v. 2.050 [38], and Haploview v. 4.1 [39] were used to perform the statistical analyses and visualize the results. Bonferroni’s correction of multiple comparisons was performed to determine the significance of the results. The criterion for significance in the GWAS was set to *p* < 7.812 × 10^−8^ (~0.05/640,000), considering that valid statistical data were obtained for approximately 570,000–640,000 SNPs. Additionally, Hardy–Weinberg equilibrium was tested using the *χ*^2^ test (*df* = 1) for genotypic distributions of SNPs that were significantly associated with the phenotypes, with values of significant deviation set to *p* = 0.05.

In the replication study for JUH samples, clinical data on the presence/absence of nausea, presence/absence of vomiting, and presence/absence of PONV (the presence/absence of nausea or vomiting) during the 24 h postoperative period were made available and used for an additional association study. Fisher’s exact test or the Cochran–Armitage trend test were conducted in analyses using all patient samples, as in the GWAS. Trend, dominant, and recessive genetic models were, again, used for the analyses. PLINK v. 1.07 [38] was used to perform the statistical analyses. The criterion for significance in the analysis was set to *p* < 0.05. Additionally, Hardy–Weinberg equilibrium was tested using the *χ*^2^ test (*df* = 1) for genotypic distributions of the candidate SNPs, with values of significant deviation set to *p* = 0.05.

### 2.5. Additional In Silico Analysis

#### 2.5.1. Power Analysis

Statistical power analyses were preliminarily performed using G*Power 3.1.3 software [40]. Power analyses for Fisher’s exact tests, with degrees of freedom set to 2, indicated that the expected power (1 minus type II error probability) was 80.0% for the type I error probability, which was set to 1.000 × 10^−7^ (close to 7.812 × 10^−8^) when risk allele frequencies for patients with nausea and/or vomiting and patients without nausea and/or vomiting were 0.2756 and 0.1000, 0.3138 and 0.1000, and 0.2729 and 0.1000, and when the sample sizes for patients with nausea and/or vomiting and patients without nausea and/or vomiting were 265 and 541, 149 and 657, and 280 and 526, respectively, in the present study. However, for the same type I error probability and sample sizes of 265 and 541, 149 and 657, and 280 and 526, the expected power decreased to 50.0% when the risk allele frequencies for patients with nausea and/or vomiting and patients without nausea and/or vomiting were 0.2478 and 0.1000, 0.2788 and 0.1000, and 0.2455 and 0.1000, respectively. Conversely, the estimated risk allele frequencies for patients with nausea and/or vomiting and patients without nausea and/or vomiting were 0.2905 and 0.1000, 0.3327 and 0.1000, and 0.2876 and 0.1000 for the same type I error probability, and sample sizes of 265 and 541, 149 and 657, and 280 and 526, respectively, were required in order to achieve 90% power. Therefore, a single analysis in the present study was expected to detect true associations with the phenotypes, with 80% statistical power for effect sizes from large to moderately medium but not small, although the exact effect size is poorly understood in cases of SNPs that significantly contribute to PONV.

#### 2.5.2. Reference of Databases

Several databases and bioinformatic tools were referenced to more thoroughly examine the candidate SNPs that may be related to human vulnerability to PONV, including the National Center for Biotechnology (NCBI) database (http://www.ncbi.nlm.nih.gov; accessed on 19 January 2023), HaploReg v. 4.1 (https://pubs.broadinstitute.org/mammals/haploreg/haploreg.php; accessed on 25 June 2023) [41], SNPinfo Web Server (https://snpinfo.niehs.nih.gov; accessed on 25 June 2023) [42], the Genotype-Tissue Expression (GTEx) portal (https://gtexportal.org/home/; accessed on 25 June 2023) [43], the PheWeb database (https://pheweb.jp/; accessed on 18 July 2023) [44], and the SIFT tool (https://sift.bii.a-star.edu.sg/; accessed on 18 July 2023). HaploReg is a tool for investigating non-coding genomic annotations at variations in haplotype blocks, such as potential regulatory SNPs at disease-associated sites [41]. The SNPinfo Web Server is a set of web-based SNP selection tools (freely available at https://snpinfo.niehs.nih.gov; accessed on 19 January 2023) where investigators can specify genes or linkage regions and select SNPs based on GWAS results, linkage disequilibrium (LD), and predicted functional characteristics of both coding and non-coding SNPs [42]. The GTEx project, an ongoing effort to create a comprehensive public resource to study tissue-specific gene expression and regulation [43], is the basis of the GTEx portal, which offers open access to data such as gene expression, quantitative trait loci, and histology images. The PheWeb database is a platform that releases GWAS summary statistics of the BioBank Japan Project (BBJ) [44]. The SIFT tool was utilized to estimate whether amino acid substitution would affect protein function based on sequence homology and the physical properties of amino acids. Furthermore, the protein structures of contactin 5 and ATPase phospholipid transporting 8B3 were predicted from the amino acid sequence (NCBI accession no. NP_001230199.1 and NP_001171473.1, respectively) by the SWISS-MODEL server (http://swissmodel.expasy.org/; accessed on 18 July 2023).

## 3. Results

### 3.1. Impact of Clinical Variables on the Incidence of PONV in Subjects Who Underwent Elective Surgery under General Anesthesia with Propofol or Desflurane

In the CIH samples, nausea, vomiting, and PONV (nausea and/or vomiting) occurred in 32.88%, 18.49%, and 34.74% of the subjects, respectively (Table 1). Prior to the GWAS, multivariate regression analysis was conducted to explore possible factors influencing the quantitative trait of the frequency of nausea among subjects characterized by various demographic and clinical data, described in Table 1. Significant associations were revealed between the variable-transformed frequency of nausea and gender (*β* = 0.1743, *p* = 0.0343), history of smoking (*β* = −0.2480, *p* = 0.0001), history of motion sickness (*β* = 0.1782, *p* = 0.0093), history of PONV (*β* = 0.3034, *p* = 0.0049), postoperative administration of narcotic drugs (*β* = 0.3383, *p* < 0.0001), frequency of pain (*β* = 0.0240, *p* = 0.0267), and duration of surgery (min) (*β* = 0.0007 *p* = 0.0152). Therefore, these variables were incorporated as covariates in the linear regression analyses for the frequency of nausea.

### 3.2. Identification of Genetic Polymorphisms Associated with PONV in All Patients Who Underwent Elective Surgery under General Anesthesia with Propofol or Desflurane

**Figure 1:**
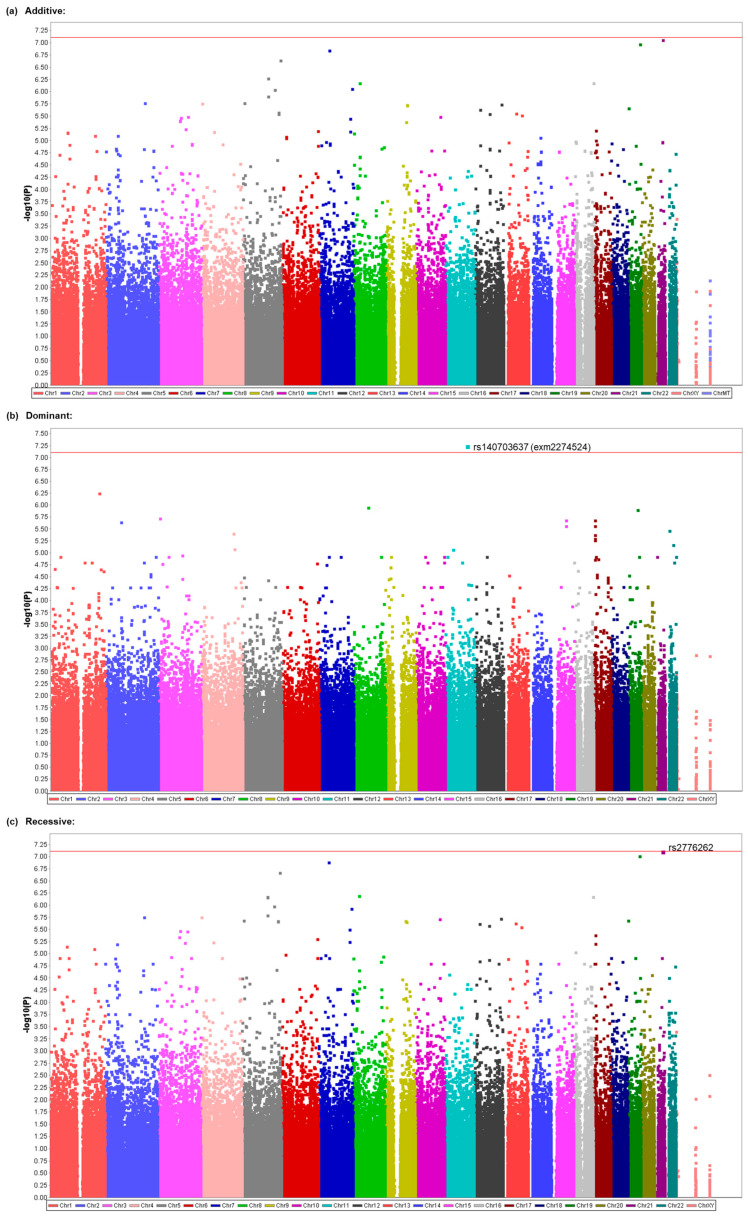
Manhattan plot as a result of the GWAS for the frequency of nausea in all patients. (**a**) Plot of the results from the additive model. (**b**) Plot of the results from the dominant model. (**c**) Plot of the results from the recessive model. The highest part of each dot represents the calculated value. The red line indicates the threshold for a significant association.

We comprehensively explored genetic variants that were associated with the presence or absence of nausea, frequency of nausea, presence or absence of vomiting, and presence or absence of PONV in a total of 806 patient subjects of CIH samples. We investigated common genetic factors for PONV by TIVA with propofol or inhalational anesthesia with desflurane. A total of 943,259 SNPs that met the quality control standards in the GWAS of all patients were examined for their relationships with the phenotypes in the trend, additive, dominant, and recessive models. No SNPs showed significant associations with the presence or absence of nausea, presence or absence of vomiting, or presence or absence of PONV (Supplementary Tables S2–S4), but significant associations were found with the frequency of nausea. Significant associations were found for the rs140703637 (exm2274524) SNP on chromosome 11 in the dominant model (*p* = 5.555 × 10^−8^; Table 2, Figure 1b) and the rs2776262 SNP on chromosome 21 in the recessive model (*p* = 7.573 × 10^−8^; Table 2, Figure 1c). Many of the examined SNPs’ computed -log10 *p*-values (observed *p*-values), which were based on the null hypothesis of a uniform distribution in the QQ plot, differed from the predicted values (Supplementary Figure S1). The values for SNPs with significant associations in Table 2 (rs140703637 and rs2776262) and other SNPs were obviously higher than the predicted values (Supplementary Figure S1b,c). However, no significant associations were found in the additive model for this phenotype (Table 2, Figure 1a). The rs140703637 SNP is located in the exon region of the contactin 5 (*CNTN5*) gene, which leads to missense mutation of the gene, and the rs2776262 SNP is located in the intron region of the *LOC100506403* gene, which is a non-coding gene that is not characterized well according to the annotation file that was supplied by manufacturer of the BeadChips or NCBI database (Table 3). As shown in Table 2, when the heterozygous and homozygous minor alleles of the rs140703637 and rs2776262 SNPs were carried, respectively, it was associated with a greater frequency of nausea during the 48 h postoperative period. None of the genotype distributions for the SNPs that were significantly associated with the phenotype significantly deviated from theoretical Hardy–Weinberg equilibrium (*χ^2^* = 0.0050, *p* = 0.9975 for rs140703637; χ^2^ = 0.2384, *p* = 0.8876 for rs2776262).

### 3.3. Identification of Genetic Polymorphisms Associated with PONV in Patients Who Underwent Elective Surgery under General Anesthesia with Propofol

**Figure 2:**
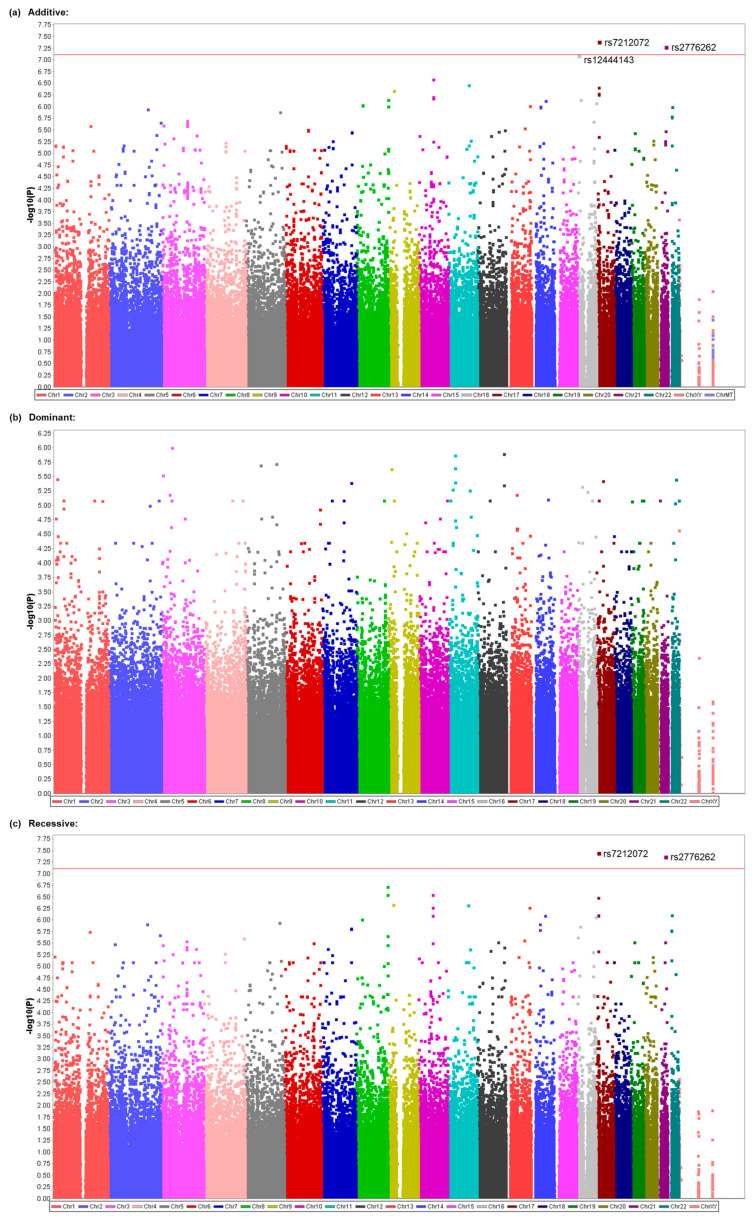
Manhattan plot as a result of the GWAS for the frequency of nausea in patients who received propofol. (**a**) Plot of the results from the additive model. (**b**) Plot of the results from the dominant model. (**c**) Plot of the results from the recessive model. The highest part of each dot represents the calculated value. The red line indicates the threshold for a significant association.

**Figure 3:**
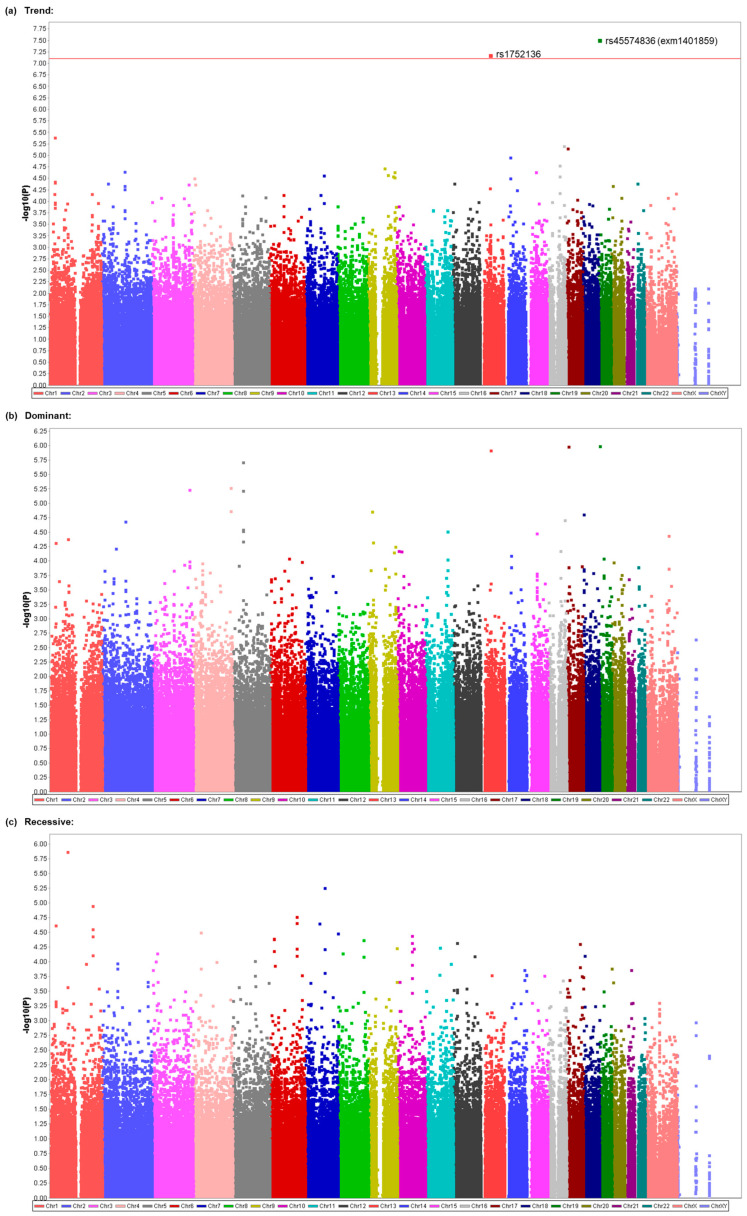
Manhattan plot as a result of the GWAS for vomiting in patients who received propofol. (**a**) Plot of the results from the trend model. (**b**) Plot of the results from the dominant model. (**c**) Plot of the results from the recessive model. The highest point of each dot represents the calculated value. The red line indicates the threshold for a significant association.

Although two kinds of anesthesia were used in patients who underwent general anesthesia at The Cancer Institute Hospital, the major type of anesthesia was TIVA by propofol (Table 1). Considering the possibility that genetic factors would more greatly contribute to the incidence of PONV without the use of volatile anesthetics compared with the use of volatile anesthetics, which are known to cause PONV more often than i.v. anesthetics, we then conducted another GWAS of the same SNPs by including only a subgroup of 442 patients who underwent general anesthesia by TIVA with propofol in CIH samples (Table 1). No SNPs showed significant associations with the presence or absence of nausea or PONV (Supplementary Tables S5 and S6), but significant associations were found with the frequency of nausea. Significant associations were found for the rs7212072 SNP on chromosome 17 (*p* = 3.919 × 10^−8^), the rs2776262 SNP on chromosome 21 (*p* = 5.028 × 10^−8^), and the rs12444143 SNP on chromosome 16 (*p* = 7.770 × 10^−8^) in the additive model (Table 3, Figure 2a). Associations were also significant for the rs7212072 SNP (*p* = 3.412 × 10^−8^) and rs2776262 SNP (*p* = 4.121 × 10^−8^) in the recessive model (Table 3, Figure 2c). However, no significant associations were found in the dominant model for this phenotype (Table 3, Figure 2b). Many of the examined SNPs’ computed -log10 *p* values (observed *p* values) differed from the predicted values (Supplementary Figure S2), and the values for SNPs with significant associations in Table 3 (rs7212072, rs2776262, and rs12444143) and other SNPs were obviously higher than the predicted values (Supplementary Figure S2a,c). Additionally, significant associations were found between the presence or absence of vomiting and the rs45574836 (exm1401859) SNP on chromosome 19 (*p* = 2.972 × 10^−8^) and rs1752136 SNP on chromosome 13 (*p* = 6.384 × 10^−8^) in the trend model (Table 4, Figure 3a). However, significant associations were not found in the dominant or recessive models in this phenotype (Table 4, Figure 3b,c). Many of the examined SNPs’ computed −log10 *p* values (observed *p* values) differed from the predicted values (Supplementary Figure S3), and the values for SNPs with significant associations in Table 4 (rs45574836 and rs1752136) and other SNPs were obviously higher than the predicted values (Supplementary Figure S3a). The rs7212072 and rs12444143 SNPs are located in intron regions of genes that encode shisa family member 6 (*SHISA6*) and RNA binding fox-1 homolog 1 (*RBFOX1*), respectively (Table 3), and the rs45574836 SNP is located in exon regions of the gene that encodes ATPase phospholipid transporting 8B3 (*ATP8B3*), which leads to missense mutation of the gene (Table 4). The rs1752136 SNP is located in intron regions of the *LOC105370198* gene, which is a non-coding gene that is not characterized well according to the annotation file that was supplied by the manufacturer of the BeadChips or NCBI database (Table 4). As shown in Table 3 and Table 4, the presence of minor alleles of the rs7212072, rs2776262, rs12444143, rs45574836, and rs1752136 SNPs was additively or homozygously associated with a greater frequency of nausea or greater incidence of vomiting during the 48 h postoperative period. None of the genotype distributions for the SNPs that were significantly associated with the phenotypes significantly deviated from the theoretical Hardy–Weinberg equilibrium (*χ*^2^ = 3.1493, *p* = 0.0760 for rs7212072; *χ*^2^ = 0.3401, *p* = 0.5598 for rs2776262; *χ*^2^ = 2.8784, *p* = 0.0898 for rs12444143; *χ*^2^ = 0.2499, *p* = 0.6171 for rs45574836; *χ*^2^ = 0.7072, *p* = 0.4004 for rs1752136).

### 3.4. Replication of Possible Association between the rs45574836 (exm1401859) SNP and Vomiting in Patients Who Underwent Gynecological Laparoscopic Surgery

The observed associations between the rs140703637, rs2776262, rs7212072, and rs12444143 SNPs and the frequency of nausea, as well as between the rs45574836 and rs1752136 SNPs and the presence of vomiting, that were identified in the GWAS of CIH samples suggest that the rs140703637, rs2776262, rs7212072, and rs12444143 SNPs were related to vulnerability to nausea, whereas the rs45574836 and rs1752136 SNPs were related to the vulnerability to vomiting in this cohort. Carriers of risk alleles of these SNPs may have presented higher vulnerability than non-carriers. To examine whether the possible difference between genotypes in the vulnerability to nausea/vomiting could be corroborated in another cohort of patients, we compared phenotypic traits that were related to PONV between genotypes of these SNPs in patients who underwent LGS (JUH samples). Clinical data on the frequency of nausea during the postoperative period were unavailable, but clinical data on the presence/absence of nausea, vomiting, and PONV were available in the JUH samples. Although the probe for the rs1752136 SNP was not included in the SNP array for genotyping in JUH samples, the probe for the rs45574836 SNP was included in the SNP array, and genotype data were available. Therefore, an additional association study for replication of the GWAS results was conducted for this SNP. The association analyses of the presence/absence of nausea, vomiting, and PONV revealed significant associations with vomiting in the trend and dominant models (*p* = 0.0239 in the trend model; *p* = 0.0313 in the dominant model; Table 5). Significant associations were also found between this SNP and PONV in the trend and dominant models (*p* = 0.0464 in trend model; *p* = 0.0289 in dominant model; Table 5). The association between this SNP and nausea was marginally significant in the dominant model (*p* = 0.0645), but no significant associations were found in the other analyses (Table 5). As observed in the GWAS of CIH samples, strong associations between this SNP and vomiting in the trend and dominant models were observed in the replication study of JUH samples (Table 5), suggesting that the presence of the minor A allele of this SNP is associated with PONV, especially vomiting. The genotype distributions for this SNP in JUH samples were 263, 67, and 3 for the G/G, G/A, and A/A genotypes, respectively, which did not significantly deviate from the theoretical Hardy–Weinberg equilibrium (*χ^2^* = 0.3158, *p* = 0.5742).

## 4. Discussion

To identify potential genetic variants that contribute to the vulnerability to PONV, we conducted a GWAS on patient subjects who underwent general anesthesia by TIVA with propofol or inhalational anesthesia with desflurane (Table 1). Our GWAS identified several potent SNPs that may possibly be associated with the frequency of nausea, including rs2776262, rs7212072, and rs12444143 in the additive and/or recessive models (Table 2 and Table 3; Figure 1 and Figure 2) and rs140703637 (exm2274524) in the dominant model (Table 2; Figure 1). Our GWAS also identified potent SNPs that may possibly be associated with the presence/absence of vomiting, including rs45574836 (exm1401859) and rs1752136 in the trend model (Table 4; Figure 3). Among them, the association between the rs45574836 (exm1401859) SNP and vomiting that was found in CIH samples was replicated in JUH samples (Table 5). The results suggested that carriers of the minor A allele of the rs45574836 SNP in the *ATP8B3* gene region that leads to a missense mutation were more vulnerable to vomiting, and, thus, tended to experience vomiting more often than noncarriers during the postoperative period (Table 4 and Table 5). Future studies with larger sample sizes are required in order to corroborate the significant results that were identified in our GWAS.

Previous studies have identified several genetic variants that are associated with phenotypes that are related to PONV [11,12,13,14]. Included in the variants that have been identified in previous candidate gene association studies are the rs33940208, rs1985242, rs10160548, and rs1176713 SNPs of the *HTR3A* gene [15,16]; the rs34236293, rs45519137, rs3758987, and -100_-102AAG deletion polymorphisms of the *HTR3B* gene [16,17,18]; the rs1800497 SNP of the *DRD2*/*ANKK1* gene [19,20]; the rs1799971 and rs9397685 SNPs of the *OPRM1* gene [21,22]; the rs3755468 SNP of the *TACR1* gene [23]; the 5-HTTLPR and rs25531 polymorphisms of the *SLC6A4* gene [24]; the rs2032582 and rs1045642 SNPs of the *ABCB1* gene [25,26,27]; the rs12208357 SNP of the *OCT1* (*SLC22A1*) gene [28]; and the rs16947, rs35742686, rs1135824, rs3892097, rs5030655, rs5030867, rs5030865, rs5030656, rs1065852, and rs5030863 SNPs of the *CYP2D6* gene [12,29,30,31]. Additionally, genetic variants identified in previous GWASs were the rs2165870 SNP of the *CHRM3* gene, the rs349358 SNP of the *KCNB2* gene, the rs1800497 SNP of the *DRD2*/*ANKK1* gene, the rs1333114 SNP of the *PTPRD* gene, and the rs11232965 SNP of the *MIR4300HG* gene [32,33,34,35]. Among these SNPs, the rs10160548 SNP of the *HTR3A* gene, the rs3758987 SNP of the *HTR3B* gene, the rs1800497 SNP of the *DRD2*/*ANKK1* gene, the rs1799971 SNP of the *OPRM1* gene, the rs3755468 SNP of the *TACR1* gene, and the rs2032582 and rs1045642 SNPs of the *ABCB1* gene were included in the SNP array that was used in the present study. However, almost none of these SNPs were even nominally significantly associated with PONV-related phenotypes (*p* > 0.05) according to our association analysis (details not shown), except for the rs10160548 SNP of the *HTR3A* gene and the rs1799971 SNP of the *OPRM1* gene, which showed some associations in patients who received propofol anesthesia (*p* = 0.03864, nausea in additive model; *p* = 0.03512, nausea in recessive model; *p* = 0.02408, PONV in additive model; and *p* = 0.004705, PONV in the recessive model for the rs10160548 SNP; *p* = 0.04958, frequency of nausea in the dominant model for the rs1799971 SNP). For the rs10160548 SNP, the results of our association analysis indicated that the minor allele was associated with a lower incidence of nausea/PONV compared with noncarriers in patients who received propofol anesthesia, which appears to be in accordance with the trend that was observed for nausea in a previous report by Lin et al. (2014) [15]. For the rs1799971 SNP, the results of our association analysis indicated that carriers of the G/G genotype were associated with more frequent nausea compared with noncarriers, which appears to be the opposite trend to the results that were reported by Lee et al. (2015), in which patients with the G/G genotype were lower on the PONV scale upon arrival to the post-anesthesia care unit [21]. These different results between the present study and previous studies might indicate the general difficulty of replicating the results of human genetic association studies, likely because of the heterogeneity of the study designs, length of the postoperative period during which PONV was recorded, the severity of PONV, the types of surgery, the types of anesthetics, and the genetic backgrounds of the subjects, among other factors.

Propofol is one of the most commonly used intravenous anesthetics, and inhalational volatile anesthetics, such as sevoflurane and desflurane, are also often used in general anesthesia. Indeed, most previous genetic association studies of PONV-related phenotypes were conducted in patients who underwent general anesthesia with these anesthetics [14,15,18,19,20,21,22,23,25,26,28,30,33,34,35]. In the present study, the subjects who were recruited for the GWAS were patients who underwent general anesthesia with either propofol or desflurane. Given that the use of volatile anesthetics, per se, is known as a strong anesthesia-related risk factor for PONV [8,9,10], one could assume that genetic factors would more greatly contribute to the incidence of PONV without the use of volatile anesthetics compared with that with the use of volatile anesthetics. From this viewpoint, we conducted two kinds of GWASs. One was performed in all subjects to investigate common genetic factors for PONV by TIVA with propofol or inhalational anesthesia with desflurane. The other was performed only in subjects who underwent general anesthesia by TIVA with propofol. As a result, two SNPs were identified to be significantly associated with the frequency of nausea in all subjects (Table 2; Figure 1), and three SNPs were identified to be significantly associated with the same phenotype in the subgroup of subjects who underwent general anesthesia by TIVA with propofol (Table 3; Figure 2), among which one SNP was common in both subject groups (Table 2 and Table 3; Figure 1 and Figure 2). Moreover, the analysis of the subgroup of subjects also identified two SNPs that were significantly associated with the incidence of vomiting (Table 4; Figure 3), which were not identified to be significantly associated with the same phenotype in the analysis of all subjects (Supplementary Table S3), although the sample size in the analysis for the subgroup with propofol was much smaller than that for all subjects (Table 1). These outcomes might suggest the possibility that different genetic variants are involved in the etiology of PONV in the case of propofol and desflurane, as well as suggesting the importance of the homogeneity of samples used in human genetic association studies.

The best candidate SNPs in the present study were rs2776262, rs140703637, rs7212072, and rs12444143 for the frequency of nausea (Table 2 and Table 3; Figure 1 and Figure 2) and rs45574836 and rs1752136 for the presence/absence of vomiting (Table 4; Figure 3). The rs2776262, rs140703637, rs7212072, rs12444143, rs45574836, and rs1752136 SNPs are located in the *LOC100506403*, *CNTN5*, *SHISA6*, *RBFOX1*, *ATP8B3*, and *LOC105370198* genes, respectively (Table 2, Table 3 and Table 4). Among them, *LOC100506403* and *LOC105370198* are non-coding genes that do not appear to have been characterized well to date. The *CNTN5*, *SHISA6*, *RBFOX1*, and *ATP8B3* genes encode contactin 5, shisa family member 6, RNA binding fox-1 homolog 1, and ATPase phospholipid transporting 8B3, respectively. According to the NCBI database, contactin 5 is a member of the immunoglobulin superfamily and contactin family, which mediate cell surface interactions during nervous system development, and biased expression in the placenta and thyroid, among others, has been observed. Likewise, shisa family member 6 is predicted to enable ionotropic glutamate receptor binding activity and to be involved in several processes, including excitatory chemical synaptic transmission, the regulation of short-term neuronal synaptic plasticity, and the regulation of signal transduction. Biased expression in the brain and endometrium, among others, has been observed. The RNA-binding fox-1 homolog 1 is known as a Fox-1 family of RNA-binding proteins that regulates tissue-specific alternative splicing in metazoa, and biased expression in the brain and heart has been observed. The ATPase phospholipid transporting 8B3 belongs to the family of P-type cation transport ATPases and the subfamily of aminophospholipid-transporting ATPases that transport phosphatidylserine and phosphatidylethanolamine from one side of a bilayer to the other. Biased expression in the testis and endometrium, among others, has been observed.

To date, none of the *LOC100506403*, *CNTN5*, *SHISA6*, *RBFOX1*, *ATP8B3*, and *LOC105370198* genes have been reported to be involved in mechanisms of nausea or vomiting. Although *Cntn5* knockout animals exhibited no behavioral abnormalities, the mice were leaner, with less body mass and lower fat percentages than wildtype animals. Their cardiovascular parameters (heart rate, blood pressure, and blood flow speed) were elevated compared with the controls [45]. According to the PheWeb database [44], the rs140703637 SNP of the *CNTN5* gene is significantly associated with Parkinson’s disease, in which the dopamine system is known to be involved in its etiology [46]; it is also known to be involved in the etiology of PONV [47]. To our knowledge, no previous reports have suggested that functional changes are caused by this nonsynonymous polymorphism. Functional changes may not be easily evaluated by structural changes in the protein, which could be predicted from each amino acid sequence by the SWISS-MODEL server (Supplementary Figure S4). However, our database search estimated that the possible impact of the amino-acid substitution from isoleucine to leucine, through a base substitution from A to C in the rs140703637 SNP, on the structure and function of the human protein is predicted to be “deleterious” according to the SIFT tool, suggesting that a dramatic change may be expected to result from this SNP. For *SHISA6* and *RBFOX1*, although no relationships with PONV have been reported, two SNPs in these genes, rs2908972 and rs10500355, respectively, were interestingly shown to be strongly associated with myopia (*p* = 5.000 × 10^−24^ for rs2908972; *p* = 2.000 × 10^−63^ for rs10500355) [48] according to the Phenotype-Genotype Integrator (PheGenI), which is available in the NCBI database. This might imply that these two genes are commonly involved in mechanisms of myopia and nausea. Although our database search in the GTEx portal [43] did not find any significant associations between the best candidate SNPs in the present study, which are mentioned above, and gene expression, the rs2256472 and rs2406801 SNPs, which were strongly linked to rs1752136 (*r^2^* ≥ 0.8) based on HaploReg v. 4.1 [41] and the SNPinfo Web Server [42], were shown to be significantly associated with the expression of two nearby genes, succinate-CoA ligase ADP-forming subunit β (*SUCLA2*) and long intergenic non-protein coding RNA 562 (*LINC00562*). This suggests that this SNP is involved in mechanisms of vomiting through the alteration of expression of these genes, although further details are unknown. A role of Atp8b3 in mouse sperm cell capacitation has been suggested [49], but the possible relationship between ATP8B3 and PONV remains unknown. Moreover, the amino acid substitution from alanine to threonine, through a base substitution from G to A in the rs45574836 SNP, seemingly caused no fundamental changes in our predicted protein structures according to the SWISS-MODEL server (Supplementary Figure S5). However, according to the PheWeb database, the rs45574836 SNP is moderately associated with constipation (*p* = 2.9 × 10^−3^), which is known as a major side effect of opioids, as well as nausea and vomiting [50]. Furthermore, the association between the rs45574836 SNP and vomiting in the CIH samples was replicated in the JUH samples in the same genetic model in the present study (Table 5), suggesting that this is a plausible candidate SNP that contributes to individual differences in the vulnerability to vomiting.

One limitation of the present study was the lack of detailed surgical records in the CIH samples; thus, no details regarding surgical procedures are described in the CIH cohort (Table 1). Given that cholecystectomy, gynecological surgery, and laparoscopic procedures have been observed as surgical risk factors for PONV in addition to other anesthesia-related predictors [8,9,10], the incidence of PONV may be influenced by specific types of surgeries in the subjects, suggesting that the strength of impact of certain types of surgeries as an environmental factor that influences PONV can be different among various surgeries. The lack of surgical information for each patient in the present study hampered our ability to conduct a GWAS in subjects with more homogeneity. In the present study, patients who underwent numerous types of surgeries were recruited, and the GWAS was conducted regardless of type of surgery in the CIH samples. With additional information regarding types of surgeries, further GWASs could be performed in particular subsets of patients, such as only in patients undergoing laparoscopic procedures, which was difficult in the present study because of the lack of such information. These limitations notwithstanding, the present study identified several SNPs that were significantly associated with nausea and vomiting. Regardless of the type of surgery, these SNPs might commonly impact PONV, and they were associated with vulnerability to PONV in our analyses of whole samples.

## 5. Conclusions

In conclusion, our GWASs revealed that the rs2776262, rs140703637, rs7212072, and rs12444143 SNPs, as well as the rs45574836 and rs1752136 SNPs, were significantly associated with the frequency of nausea and presence/absence of vomiting, respectively, during the postoperative period in patients who underwent elective surgery under general anesthesia with propofol or desflurane. The association between the rs45574836 SNP and vomiting was replicated in patients who underwent laparoscopic gynecological surgery. Although the present results need to be corroborated by more research with larger sample sizes and in other populations, these findings indicate that these SNPs in the *LOC100506403*, *CNTN5*, *SHISA6*, *RBFOX1*, *ATP8B3*, and *LOC105370198* gene regions could serve as markers that predict the vulnerability to PONV.

## Figures and Tables

**Table 1:** Demographic and clinical data of patient subjects for the GWAS.

Demographic Data:	*n*	Minimum	Maximum	Mean	SD	Median
Gender						
male	432					
female	374					
Age [years]	806	23	93	58.80	13.25	60.00
Height [cm]	806	140.8	184.9	162.39	8.16	161.90
Weight [kg]	806	31.5	109.1	59.01	11.15	57.65
Body mass index (BMI) [kg/m^2^]	806	14.46	39.05	22.29	3.39	22.02
History of smoking	806					
absence	453					
presence	353					
Frequency of alcohol drinking per week	806	0	7	2.65	7.00	1.00
History of motion sickness	806					
absence	495					
presence	311					
History of PONV	806					
absence	722					
presence	84					
**Surgery and clinical data for postoperative period:**	***n***	**Minimum**	**Maximum**	**Mean**	**SD**	**Median**
Duration of anaesthesia [min]	806	30	1050	258.62	1050.00	214.50
Duration of surgery [min]	806	4	977	203.34	977.00	163.00
Type of anesthesia	806					
TIVA (propofol)	442					
inhalational anesthesia (desflurane)	364					
Total dose of remifentanil [μg]	806	0	24,300	3135.61	24,300.00	2500.00
Total dose of fentanyl [μg]	806	0	800	198.26	800.00	200.00
Postoperative administration of narcotic drugs	806					
absence	427					
presence	379					
Postoperative administration of opioids including pentazocine	806					
absence	340					
presence	466					
Experience of pain	806					
absence	250					
presence	556					
Frequency of pain	806	0	15	2.90	15.00	2.00
**PONV:**	***n***	**Minimum**	**Maximum**	**Mean**	**SD**	**Median**
Nausea	806					
absence	541					
presence	265					
Frequency of nausea	806	0	10	0.83	10.00	0.00
Vomiting	806					
absence	657					
presence	149					
PONV	806					
absence	526					
presence	280					

**Table 2:** Top 20 candidate SNPs selected from the GWAS for the frequency of nausea in all patients.

Model	Rank	CHR	SNP	Position	*p*	Related Gene	Genotype (Patients)			Genotype (Mean)		
							A/A	A/B	B/B	A/A	A/B	B/B
Additive	1	21	rs2776262	36940158	0.00000008533	(*LOC100506403*)	2	95	709	4.145	0.313	0.556
Additive	2	19	rs12609817	53859182	0.0000001055		4	109	693	2.882	0.486	0.531
Additive	3	7	rs921634	47872845	0.0000001393	*PKD1L1*	9	145	652	2.293	0.479	0.525
Additive	4	5	rs1587176	174851190	0.0000002228		5	172	628	2.761	0.552	0.515
Additive	5	5	rs10061408	117292776	0.0000005159		6	109	691	2.446	0.61	0.508
Additive	6	5	rs10071777	117286831	0.000000524		6	109	690	2.446	0.61	0.509
Additive	7	8	rs6558049	28093041	0.0000006488		5	109	692	2.747	0.508	0.525
Additive	8	16	rs7192373	90032149	0.0000006522	*DEF8*	2	58	746	3.612	0.588	0.524
Additive	9	7	rs17173793	153523345	0.0000008597		2	111	691	3.766	0.707	0.499
Additive	10	5	rs13159091	148067337	0.0000008984		2	100	704	3.808	0.638	0.513
Additive	11	5	rs17414326	117297258	0.000001209		2	68	736	3.646	0.708	0.512
Additive	12	5	rs1157652	8565638	0.000001661		21	193	592	1.572	0.543	0.497
Additive	13	2	rs3112976	180440437	0.000001666	*ZNF385B*	2	118	686	3.449	0.595	0.518
Additive	14	4	exm2265817	5023112	0.000001687		2	64	740	3.808	0.57	0.525
Additive	14	4	rs10937615	5023112	0.000001687		2	64	740	3.808	0.57	0.525
Additive	16	12	exm-rs11057830	125307053	0.000001762	*SCARB1*	3	120	683	2.986	0.523	0.528
Additive	17	9	exm-rs755109	100696203	0.000001821	*HEMGN*	13	176	617	1.713	0.542	0.51
Additive	18	9	rs1475696	100691397	0.000001857	*HEMGN*	13	175	618	1.713	0.54	0.511
Additive	19	19	rs11084950	2657397	0.000002102	*GNG7*	2	104	700	3.766	0.503	0.532
Additive	20	12	rs2137547	26261873	0.000002264		3	91	712	3.018	0.603	0.517
Dominant	1	11	exm2274524	100221580	0.00000005555 *	*CNTN5*	0	4	801	NA	2.913	0.52
Dominant	2	1	exm1762808	220823972	0.0000005544	*MARK1*	0	2	802	NA	3.766	0.53
Dominant	3	8	rs10087234	68735313	0.000001101		0	7	799	NA	2.205	0.522
Dominant	4	19	exm1473939	42937953	0.000001221	*CXCL17*	1	7	798	0	2.64	0.519
Dominant	5	3	rs164464	8749012	0.000001845		0	2	803	NA	3.553	0.53
Dominant	6	3	rs241055	8738790	0.000001855		0	2	804	NA	3.553	0.529
Dominant	6	3	rs12497498	8751490	0.000001855		0	2	804	NA	3.553	0.529
Dominant	6	3	rs17049459	8751984	0.000001855		0	2	804	NA	3.553	0.529
Dominant	9	15	rs16948440	65255168	0.000002007		0	2	790	NA	3.612	0.524
Dominant	9	17	rs222843	7145981	0.000002007		121	373	311	0.438	0.384	0.759
Dominant	11	2	rs2075225	71063066	0.00000221		106	373	326	0.451	0.392	0.727
Dominant	12	17	exm1286317	7163739	0.000002647	*CLDN7*	121	374	311	0.438	0.385	0.756
Dominant	13	15	rs34636936	65297261	0.000002663	*MTFMT*	0	2	804	NA	3.612	0.529
Dominant	14	22	rs1210829	20308800	0.000003367		21	211	569	0.523	0.818	0.437
Dominant	15	4	rs2204206	149713872	0.000003862		207	374	225	0.41	0.471	0.761
Dominant	16	17	rs222835	7134129	0.000004137	*DVL2*	120	362	324	0.481	0.365	0.749
Dominant	17	17	rs222837	7132556	0.000004902	*DVL2*	118	363	325	0.477	0.368	0.746
Dominant	18	17	rs739669	7122377	0.000005338	*DLG4*	118	362	326	0.477	0.369	0.744
Dominant	19	22	exm2010161	38474406	0.000006687	*SLC16A8*	0	2	804	NA	3.278	0.53
Dominant	20	4	rs11734518	152213113	0.000008271		107	377	322	0.705	0.642	0.356
Recessive	1	21	rs2776262	36940158	0.00000007573 *	(*LOC100506403*)	2	95	709	4.145	0.313	0.556
Recessive	2	19	rs12609817	53859182	0.00000009569		4	109	693	2.882	0.486	0.531
Recessive	3	7	rs921634	47872845	0.0000001274	*PKD1L1*	9	145	652	2.293	0.479	0.525
Recessive	4	5	rs1587176	174851190	0.0000002107		5	172	628	2.761	0.552	0.515
Recessive	5	8	rs6558049	28093041	0.0000006248		5	109	692	2.747	0.508	0.525
Recessive	6	5	rs10061408	117292776	0.0000006556		6	109	691	2.446	0.61	0.508
Recessive	7	16	rs7192373	90032149	0.0000006607	*DEF8*	2	58	746	3.612	0.588	0.524
Recessive	8	5	rs10071777	117286831	0.0000006649		6	109	690	2.446	0.61	0.509
Recessive	9	5	rs13159091	148067337	0.000001017		2	100	704	3.808	0.638	0.513
Recessive	10	7	rs17173793	153523345	0.00000114		2	111	691	3.766	0.707	0.499
Recessive	11	5	rs17414326	117297258	0.000001587		2	68	736	3.646	0.708	0.512
Recessive	12	4	exm2265817	5023112	0.000001705		2	64	740	3.808	0.57	0.525
Recessive	12	4	rs10937615	5023112	0.000001705		2	64	740	3.808	0.57	0.525
Recessive	14	2	rs3112976	180440437	0.00000171	*ZNF385B*	2	118	686	3.449	0.595	0.518
Recessive	15	12	exm-rs11057830	125307053	0.00000185	*SCARB1*	3	120	683	2.986	0.523	0.528
Recessive	16	10	rs1342273	111973339	0.00000187	*MXI1*	12	159	635	1.815	0.41	0.544
Recessive	17	5	rs1157652	8565638	0.000001997		21	193	592	1.572	0.543	0.497
Recessive	18	19	rs11084950	2657397	0.00000201	*GNG7*	2	104	700	3.766	0.503	0.532
Recessive	19	5	rs2591580	165406607	0.000002074		12	165	629	1.801	0.438	0.538
Recessive	20	9	rs10115047	96631287	0.000002086		25	238	543	1.457	0.47	0.523

Model, the genetic model in which candidate SNPs were selected by the GWAS; CHR, chromosome number; Position, chromosomal position (bp); *, significant after Bonferroni correction for multiple comparisons (*p* < 7.812 × 10^−8^); Related gene, the nearest gene from the SNP site; A/A, homozygote for the minor allele for each SNP; A/B, heterozygote for each SNP; B/B, homozygote for the major allele for each SNP; NA, not available.

**Table 3:** Top 20 candidate SNPs selected from the GWAS for the frequency of nausea in patients who received propofol.

Model	Rank	CHR	SNP	Position	*p*	Related Gene	Genotype (Patients)			Genotype (Mean)		
							A/A	A/B	B/B	A/A	A/B	B/B
Additive	1	17	rs7212072	11310500	0.00000003919 *	*SHISA6*	13	158	271	2.02	0.523	0.504
Additive	2	21	rs2776262	36940158	0.00000005028 *	(*LOC100506403*)	2	45	395	4.145	0.342	0.562
Additive	3	16	rs12444143	6071910	0.0000000777 *	*RBFOX1*	84	197	161	0.981	0.572	0.312
Additive	4	10	rs2499891	66680444	0.000000254		21	173	248	1.499	0.586	0.454
Additive	5	11	rs7110244	95910507	0.0000003371	*MAML2*	3	61	378	3.262	0.71	0.509
Additive	6	17	rs1513743	11306268	0.000000375	*SHISA6*	14	159	269	1.875	0.52	0.508
Additive	7	9	rs1937955	25721500	0.0000004509		2	60	380	3.808	0.559	0.538
Additive	8	17	rs4293419	11306043	0.0000005146	*SHISA6*	3	96	343	3.413	0.646	0.505
Additive	9	17	rs1034899	11306660	0.000000536	*SHISA6*	3	96	342	3.413	0.646	0.507
Additive	10	10	rs7911209	66726865	0.0000006068		20	176	245	1.501	0.573	0.467
Additive	11	10	rs10733810	66723680	0.0000006447		20	168	254	1.501	0.595	0.455
Additive	12	8	rs1735176	146074281	0.0000006883		18	164	259	1.733	0.443	0.547
Additive	13	16	rs11649132	17032787	0.0000006937		15	138	289	1.587	0.601	0.48
Additive	14	14	rs1256520	65737193	0.0000007361		2	81	359	4.162	0.606	0.524
Additive	14	14	rs1256526	65739905	0.0000007361		2	81	359	4.162	0.606	0.524
Additive	16	16	rs7192373	90032149	0.0000008101	*DEF8*	2	29	411	3.612	0.677	0.532
Additive	17	8	rs2322976	28106141	0.0000009019		2	57	383	3.808	0.601	0.532
Additive	18	8	rs6558049	28093041	0.0000009166		2	59	381	3.808	0.58	0.534
Additive	19	13	rs816958	108522895	0.0000009365		14	96	331	1.878	0.466	0.527
Additive	20	8	exm734131	146076708	0.0000009716	*COMMD5*	18	164	260	1.687	0.448	0.545
Additive	20	8	rs1209879	146076708	0.0000009716	*COMMD5*	18	164	260	1.687	0.448	0.545
Dominant	1	3	rs13100791	49641049	0.0000009768	*BSN*	0	4	438	NA	2.629	0.536
Dominant	1	3	exm315855	49737954	0.0000009768	*RNF123*	0	4	438	NA	2.629	0.536
Dominant	1	3	exm316496	49869455	0.0000009768	*TRAIP*	0	4	438	NA	2.629	0.536
Dominant	4	12	exm1046926	123340542	0.000001253	*HIP1R*	0	2	440	NA	3.808	0.541
Dominant	5	11	rs7933966	32875597	0.000001333	*PRRG4*	94	224	124	0.501	0.379	0.915
Dominant	5	11	exm-rs10767971	32895664	0.000001333		94	224	124	0.501	0.379	0.915
Dominant	5	11	rs10767971	32895664	0.000001333		94	224	124	0.501	0.379	0.915
Dominant	8	5	rs252110	141339522	0.000001858		0	8	433	NA	2.209	0.523
Dominant	9	5	rs34221525	68798118	0.000001959	*OCLN*	0	7	435	NA	2.165	0.529
Dominant	10	11	rs4755454	32903263	0.000002224		96	225	121	0.49	0.392	0.911
Dominant	11	9	rs17725257	16216102	0.000002292		0	6	436	NA	2.427	0.53
Dominant	12	3	rs241055	8738790	0.000002929		0	2	440	NA	3.553	0.542
Dominant	12	3	rs164464	8749012	0.000002929		0	2	440	NA	3.553	0.542
Dominant	12	3	rs12497498	8751490	0.000002929		0	2	440	NA	3.553	0.542
Dominant	12	3	rs17049459	8751984	0.000002929		0	2	440	NA	3.553	0.542
Dominant	16	1	rs198412	11900437	0.000003433	*CLCN6,NPPA-AS1*	0	4	438	NA	2.964	0.533
Dominant	17	22	exm2010161	38474406	0.000003506	*SLC16A8*	0	2	440	NA	3.278	0.543
Dominant	18	17	rs17780388	30606301	0.000003661	*RHBDL3*	0	5	437	NA	2.671	0.531
Dominant	19	11	rs197697	32834416	0.000003879		92	224	126	0.503	0.383	0.901
Dominant	20	7	rs1982436	134470632	0.000003965	*CALD1*	10	128	304	0.414	0.918	0.407
Recessive	1	17	rs7212072	11310500	0.00000003412 *	*SHISA6*	13	158	271	2.02	0.523	0.504
Recessive	2	21	rs2776262	36940158	0.00000004121 *	(*LOC100506403*)	2	45	395	4.145	0.342	0.562
Recessive	3	8	rs1735176	146074281	0.0000001881		18	164	259	1.733	0.443	0.547
Recessive	4	10	rs2499891	66680444	0.0000002799		21	173	248	1.499	0.586	0.454
Recessive	5	8	exm734131	146076708	0.0000002805	*COMMD5*	18	164	260	1.687	0.448	0.545
Recessive	5	8	rs1209879	146076708	0.0000002805	*COMMD5*	18	164	260	1.687	0.448	0.545
Recessive	7	17	rs1513743	11306268	0.0000003181	*SHISA6*	14	159	269	1.875	0.52	0.508
Recessive	8	9	rs1937955	25721500	0.0000004615		2	60	380	3.808	0.559	0.538
Recessive	9	11	rs7110244	95910507	0.0000004699	*MAML2*	3	61	378	3.262	0.71	0.509
Recessive	10	13	rs816958	108522895	0.000000523		14	96	331	1.878	0.466	0.527
Recessive	11	10	rs7911209	66726865	0.0000005299		20	176	245	1.501	0.573	0.467
Recessive	12	17	rs4293419	11306043	0.0000007621	*SHISA6*	3	96	343	3.413	0.646	0.505
Recessive	13	22	rs8142156	19692751	0.0000007685		4	64	374	2.599	0.367	0.566
Recessive	13	22	rs9618670	19694333	0.0000007685		4	63	375	2.599	0.373	0.564
Recessive	15	14	rs1256520	65737193	0.0000007818		2	81	359	4.162	0.606	0.524
Recessive	15	14	rs1256526	65739905	0.0000007818		2	81	359	4.162	0.606	0.524
Recessive	17	10	rs10733810	66723680	0.0000007878		20	168	254	1.501	0.595	0.455
Recessive	18	17	rs1034899	11306660	0.0000007904	*SHISA6*	3	96	342	3.413	0.646	0.507
Recessive	19	16	rs7192373	90032149	0.0000008464	*DEF8*	2	29	411	3.612	0.677	0.532
Recessive	20	8	rs6558049	28093041	0.0000009534		2	59	381	3.808	0.58	0.534
Recessive	20	8	rs2322976	28106141	0.0000009534		2	57	383	3.808	0.601	0.532

Model, the genetic model in which candidate SNPs were selected by the GWAS; CHR, chromosome number; Position, chromosomal position (bp); *, significant after Bonferroni correction of multiple comparisons (*p* < 7.812 × 10^−8^); Related gene, the nearest gene from the SNP site; A/A, homozygote for the minor allele in each SNP; A/B, heterozygote in each SNP; B/B, homozygote for the major allele in each SNP; NA, not available.

**Table 4:** Top 20 candidate SNPs selected from the GWAS for vomiting in patients who received propofol.

Model	Rank	CHR	SNP	Position	*p*	Related Gene	Genotype (Vomiting +)			Genotype (Vomiting −)		
							A/A	A/B	B/B	A/A	A/B	B/B
Trend	1	19	exm1401859	1806667	0.00000002972 *	*ATP8B3*	4	31	62	1	44	300
Trend	2	13	rs1752136	48726219	0.00000006384 *	(*LOC105370198*)	0	20	77	0	14	331
Trend	3	1	rs6675501	24672641	0.000003994	*GRHL3*	42	41	14	73	165	107
Trend	4	16	rs16954219	80925781	0.000006147		4	43	50	4	83	258
Trend	5	17	rs16944600	11245099	0.000006816	*SHISA6*	4	23	70	27	167	151
Trend	6	14	rs10438059	33026271	0.00001081	*AKAP6*	13	48	36	16	123	205
Trend	7	16	rs12928123	61178279	0.00001632		5	33	59	5	60	280
Trend	7	16	rs9929283	61182342	0.00001632		5	33	59	5	60	280
Trend	9	9	rs10867734	83933198	0.00001865		2	14	81	0	16	329
Trend	10	2	exm-rs6726639	112753097	0.00002221	*MERTK*	31	46	15	65	132	131
Trend	11	15	rs765	48035685	0.00002252	*SEMA6D*	7	49	41	11	106	228
Trend	12	9	rs7850697	131095112	0.00002275	*COQ4*	2	41	54	38	189	118
Trend	12	9	rs7030121	131101919	0.00002275		2	41	54	38	189	118
Trend	14	9	rs12684445	98805260	0.00002621		11	47	39	14	117	214
Trend	15	7	rs12704714	93930713	0.00002643		32	39	26	42	164	138
Trend	16	16	rs12445491	61145947	0.00002755		8	30	59	6	66	273
Trend	17	9	rs306772	124092355	0.00002782	*GSN*	1	32	64	1	51	293
Trend	18	9	rs2240960	131039250	0.00002911	*SWI5*	4	37	56	39	186	118
Trend	19	4	rs2086431	9509791	0.00003047		4	28	65	3	49	293
Trend	20	14	rs17098983	32868873	0.00003088	*AKAP6*	0	20	77	19	128	198
Dominant	1	19	exm1401859	1806667	0.0000009938	*ATP8B3*	4	31	62	1	44	300
Dominant	2	17	rs16944600	11245099	0.000001015	*SHISA6*	4	23	70	27	167	151
Dominant	3	13	rs1752136	48726219	0.000001177		0	20	77	0	14	331
Dominant	4	5	rs7715247	50318550	0.000001902		7	22	68	31	167	147
Dominant	5	4	rs11946898	181926518	0.000005267		23	62	12	65	157	123
Dominant	6	3	rs1524511	179642067	0.000005693	*PEX5L*	10	53	34	23	111	211
Dominant	7	5	rs622304	50279857	0.000005922		7	20	70	27	159	159
Dominant	8	4	rs12642493	181933056	0.00001346		23	60	14	64	153	128
Dominant	9	9	rs3808657	19127877	0.00001358		11	24	61	47	167	131
Dominant	10	18	rs8093227	2078441	0.00001524		11	62	24	34	140	171
Dominant	11	16	rs16954219	80925781	0.00001919		4	43	50	4	83	258
Dominant	12	2	exm-rs6726639	112753097	0.00002021	*MERTK*	31	46	15	65	132	131
Dominant	13	5	rs250216	50281358	0.00002777		4	21	72	19	152	174
Dominant	14	5	rs13175573	50297517	0.00002949		5	23	69	22	161	162
Dominant	15	11	rs624584	107344724	0.00003038		4	12	81	12	122	211
Dominant	16	15	rs765	48035685	0.00003277	*SEMA6D*	7	49	41	11	106	228
Dominant	17	23	rs12688309	113796171	0.00003574		0	8	81	5	79	199
Dominant	18	1	rs12027987	85206774	0.00004063		8	31	58	53	167	125
Dominant	19	5	rs12659587	50269410	0.00004483		4	21	72	19	150	176
Dominant	20	9	rs9792672	23087926	0.00004684		1	4	92	4	73	268
Recessive	1	1	rs1195866	81678254	0.00000133		14	28	55	5	117	219
Recessive	2	7	rs12704714	93930713	0.000005402		32	39	26	42	164	138
Recessive	3	1	rs734591	203449615	0.00001109	*PRELP*	43	29	25	72	174	99
Recessive	4	6	rs4897427	130810954	0.00001688		29	34	33	38	177	130
Recessive	5	6	rs9492605	130796396	0.00002139		24	31	42	27	154	162
Recessive	6	7	rs2904188	68504050	0.00002176		11	21	65	4	96	245
Recessive	6	7	rs2869745	68539977	0.00002176		11	22	64	4	98	243
Recessive	8	1	rs6675501	24672641	0.00002366	*GRHL3*	42	41	14	73	165	107
Recessive	9	1	rs880878	203435718	0.00002728		43	30	24	76	172	97
Recessive	10	4	rs16994732	38717953	0.00003138		8	27	62	1	89	255
Recessive	11	7	rs12698219	158145606	0.0000321	*PTPRN2*	9	18	70	2	65	278
Recessive	12	10	rs7895191	70221267	0.00003529	*DNA2*	0	46	51	41	143	161
Recessive	12	10	rs12220316	70230840	0.00003529	*DNA2*	0	46	51	41	143	161
Recessive	14	1	rs3766902	203478370	0.00003617		39	36	22	65	158	122
Recessive	15	6	rs4395717	20826281	0.00003964	*CDKAL1*	7	59	30	89	174	82
Recessive	16	6	rs4077405	20876683	0.00004018	*CDKAL1*	7	60	30	88	174	83
Recessive	17	8	exm719103	124440174	0.00004181	*WDYHV1*	28	44	25	38	181	126
Recessive	17	8	rs6999234	124440174	0.00004181	*WDYHV1*	28	44	25	38	181	126
Recessive	17	8	exm719120	124448736	0.00004181	*WDYHV1*	28	44	25	38	181	126
Recessive	17	8	rs7014678	124448736	0.00004181	*WDYHV1*	28	44	25	38	181	126
Recessive	17	8	exm719122	124448804	0.00004181	*WDYHV1*	28	44	25	38	181	126
Recessive	17	8	exm719125	124449466	0.00004181	*WDYHV1*	28	44	25	38	181	126
Recessive	17	8	rs3824250	124449466	0.00004181	*WDYHV1*	28	44	25	38	181	126
Recessive	17	8	rs13269287	124453662	0.00004181	*WDYHV1*	28	44	25	38	181	126
Recessive	17	8	rs7822061	124456727	0.00004181		28	44	25	38	181	126

Model, the genetic model in which candidate SNPs were selected by the GWAS; CHR, chromosome number; Position, chromosomal position (bp); *, significant after Bonferroni correction of multiple comparisons (*p* < 7.812 × 10^−8^); Related gene, the nearest gene from the SNP site; A/A, homozygote for the minor allele for each SNP; A/B, heterozygote for each SNP; B/B, homozygote for the major allele for each SNP.

**Table 5:** Results of the replication study for the rs45574836 (exm1401859) SNP, selected in the GWAS for vomiting in patients who received propofol.

Phenotype (+/−)	GWAS						Replication Study					
	Genotype			*p*	*p*	*p*	Genotype			*p*	*p*	*p*
	G/G	G/A	A/A	(Trend)	(Dominant)	(Recessive)	G/G	G/A	A/A	(Trend)	(Dominant)	(Recessive)
Nausea (−)	252	39	2	0.001456	0.002497	0.3409	236	54	3	0.1010	0.06452	1
Nausea (+)	110	36	3				27	13	0			
Vomiting (−)	300	44	1	2.972 × 10^−8 †^	9.938 × 10^−7^	0.009167	253	59	3	0.02389 *	0.03131 *	1
Vomiting (+)	62	31	4				10	8	0			
PONV (−)	244	37	1	0.0003522	0.001215	0.05993	234	52	3	0.04636 *	0.02885 *	1
PONV (+)	118	38	4				29	15	0			

^†^, significant in GWAS (*p* < 7.812 × 10^−8^); *, significant in replication study (*p* < 0.05).

## Data Availability

The data that are presented in this study are available upon request from the corresponding author.

## Supplementary Materials

The following supporting information can be downloaded at: https://www.mdpi.com/article/10.3390/cancers15194729/s1. Table S1. Demographic and clinical data of patient subjects for the replication study. Table S2. Top 20 candidate SNPs selected from the GWAS for nausea in all patients. Table S3. Top 20 candidate SNPs selected from the GWAS for vomiting in all patients. Table S4. Top 20 candidate SNPs selected from the GWAS for PONV in all patients. Table S5. Top 20 candidate SNPs selected from the GWAS for nausea in patients who received propofol. Table S6. Top 20 candidate SNPs selected from the GWAS for PONV in patients who received propofol. Figure S1. Log quantile-quantile (QQ) *p*-value plot as a result of the GWAS for the frequency of nausea in all patients. Figure S2. Log quantile-quantile (QQ) *p*-value plot as a result of the GWAS for the frequency of nausea in patients with propofol. Figure S3. Log quantile-quantile (QQ) *p*-value plot as a result of the GWAS for vomiting in patients who received propofol. Figure S4. Protein structures of contactin 5 predicted from amino acid sequence (NCBI accession no. NP_001230199.1). Figure S5. Protein structures of ATPase phospholipid transporting 8B3 predicted from amino acid sequence (NCBI accession no. NP_001171473.1).
