# Supplementary material for: Genome-Wide Association Study Identifies Novel Candidate Variants Associated with Postoperative Nausea and Vomiting
Source: Cancers (Basel). 2023 Sep 26;15(19):4729. doi: 10.3390/cancers15194729 (PMC10571790; doi:10.3390/cancers15194729)
Supplement: Supplementary file 1 [file cancers-15-04729-s001.zip › cancers-2580221-supplementary.pdf]

cancers

MDPI

Supplementary Materials

Genome-Wide Association Study Identifies Novel Candidate Variants Associated with Postoperative Nausea and Vomiting

Daisuke Nishizawa [1,†], Ryozo Morino [2,†], Rie Inoue [1,3], Seii Ohka [1], Shinya Kasai [1], Junko Hasegawa [1], Yuko Ebata [1], Kyoko Nakayama [1], Hiroyuki Sumikura [3], Masakazu Hayashida [1,3], Miyuki Yokota [4,5] and Kazutaka Ikeda [1,*]

Table S1. Demographic and clinical data of patient subjects for the replication study.

Demographic data:	*n*	Minimum	Maximum	Mean	SD	Median
Gender						
male	0					
female	333					
Age [years]	333	21	69	39.20	8.60	40.00
Height [cm]	333	141.3	174.5	159.82	5.57	160.00
Weight [kg]	333	37.45	82.9	54.55	8.22	53.55
Body mass index (BMI) [kg/m^2]	333	15.10	29.70	21.36	3.09	20.90
Duration of surgery [min]	333	26	303	128.90	45.20	124.00
Duration of anaesthesia [min]	333	69	328	175.10	48.20	171.00
Total dose of propofol [mg]	333	0	1850	854.10	302.20	800.00
Average remifentanil infusion rate [µg/kg/min]	333	0.022	0.713	0.30	0.07	0.30
Frequency of acetaminophen administration	333	1	4	2.9	0.6	3
PCA fentanyl consumption (µg/kg)	333	0	20.88	5.53	4.44	4.54
Total postoperative fentanyl administration (µg/kg)	333	3.92	25.39	9.73	4.51	8.79
Incidence of PONV						
absence	289					
presence	44					
Administration of resque analgesic						
absence	306					
presence	27					
Average pain score of numerical rating scale	333	0	6	1.83	1.15	1.70

Table S2. Top 20 candidate SNPs selected from the GWAS for nausea in all patients.

Model	Rank	CHR	SNP	Position	*p*	Related gene	Genotype (Nausea +)			Genotype (Nausea -)		
							A/A	A/B	B/B	A/A	A/B	B/B
Trend	1	21	rs2836505	39918553	0.000004044	*ERG*	16	91	158	57	258	226
Trend	2	16	rs9925934	17028247	0.000005621		1	65	199	3	60	478
Trend	3	20	rs6021693	50694287	0.000006882		42	154	69	59	247	235
Trend	4	16	rs6498643	17050874	0.000008058		2	71	192	5	68	467
Trend	5	5	rs7719723	2137758	0.00001176		27	126	112	122	250	169
Trend	6	20	rs6017227	42593220	0.00001231	*TOX2*	0	39	226	0	30	511
Trend	7	5	rs1560639	173609274	0.00001324		11	81	173	11	99	431
Trend	8	17	rs1978136	32377698	0.00001458	*ACCN1*	57	138	70	68	258	215
Trend	9	17	exm1284008	5404011	0.00002298	*LOC728392*	8	78	179	5	101	435
Trend	10	13	rs9549763	114052470	0.00002432		0	50	215	22	146	373
Trend	11	7	rs1982436	134470632	0.00002639	*CALD1*	9	98	158	9	131	401
Trend	12	10	rs7073770	15417537	0.00002681		1	12	252	1	84	456
Trend	13	13	rs645833	48724256	0.00002815		0	29	236	0	19	522
Trend	14	23	rs7879223	95721436	0.00002819		0	12	127	0	1	234
Trend	15	17	rs11655926	35243370	0.00002833		24	118	123	29	176	336
Trend	16	12	rs6488519	12532984	0.00003117	*LOH12CR1*	6	43	216	1	49	491
Trend	17	1	exm2250857	230469505	0.00003232	*PGBD5*	80	140	45	113	268	160

Trend	18	15	rs8032900	39767878	0.00003274		6	76	183	7	88	446
Trend	19	13	rs612938	103347591	0.00003482		2	57	205	0	63	477
Trend	20	2	rs10931008	183238919	0.00003532	*PDE1A*	38	131	96	132	276	133
Dominant	1	20	rs6021693	50694287	0.000001494		42	154	69	59	247	235
Dominant	2	21	rs2836505	39918553	0.000002134	*ERG*	16	91	158	57	258	226
Dominant	3	16	rs6498643	17050874	0.000002438		2	71	192	5	68	467
Dominant	4	8	rs17296667	125296872	0.000002821		2	85	178	9	87	445
Dominant	5	16	rs9925934	17028247	0.000003257		1	65	199	3	60	478
Dominant	6	10	rs7073770	15417537	0.000003458		1	12	252	1	84	456
Dominant	7	10	rs17102809	123728523	0.00000427	*NSMCE4A*	19	136	110	43	179	318
Dominant	8	5	rs1560639	173609274	0.0000142		11	81	173	11	99	431
Dominant	9	1	rs10900553	204108163	0.00001572	*ETNK2*	32	118	115	87	303	151
Dominant	10	1	rs2970580	212097521	0.00001762		5	39	221	12	154	375
Dominant	11	20	rs6057649	29960458	0.00001829	*DEFB118*	20	137	108	37	196	308
Dominant	11	20	rs6087445	29990252	0.00001829		21	136	108	38	195	308
Dominant	11	20	rs2376858	30003350	0.00001829		21	136	108	38	195	308
Dominant	11	20	rs2180566	30018854	0.00001829		21	136	108	39	194	308
Dominant	11	20	rs6088215	30021789	0.00001829		21	136	108	38	195	308
Dominant	16	1	rs16839692	240438307	0.00002474	*FMN2*	68	145	52	107	251	183
Dominant	17	20	rs6017227	42593220	0.00002506	*TOX2*	0	39	226	0	30	511
Dominant	18	20	rs709045	29965014	0.00002622	*DEFB119*	20	136	109	37	196	308

Dominant	18	20	exm2272889	29968863	0.00002622	*DEFB119*	20	136	109	37	196	308
Dominant	18	20	rs4911274	29968863	0.00002622	*DEFB119*	20	136	109	37	196	308
Dominant	18	20	rs2206381	29978216	0.00002622	*DEFB119*	20	136	109	37	196	308
Dominant	18	20	rs765736	29981907	0.00002622		20	136	109	37	196	308
Recessive	1	6	rs9403521	143994199	0.000002564	*PHACTR2*	11	138	116	80	241	220
Recessive	2	22	rs4823376	45171729	0.000002832	*PRR5-ARHGAP8,ARHGAP8*	9	117	139	72	191	278
Recessive	3	6	rs9403523	144004981	0.000003427	*PHACTR2*	13	139	113	86	241	214
Recessive	4	13	rs1782805	40946993	0.00000906	*TTL*	19	84	162	6	182	353
Recessive	5	5	rs7719723	2137758	0.00001246		27	126	112	122	250	169
Recessive	6	10	rs1125527	123019616	0.00001714		78	122	65	87	282	172
Recessive	7	1	rs1539206	212580922	0.0000329	*TMEM206*	79	114	72	91	267	183
Recessive	8	11	rs7122607	109641652	0.00003487		2	86	177	37	176	328
Recessive	9	11	rs989695	109640972	0.00003542		2	83	179	37	175	329
Recessive	10	7	rs4141275	29079870	0.00003835	*CPVL*	81	124	60	94	289	158
Recessive	11	22	rs136723	45916679	0.00003852	*FBLN1*	29	95	141	18	206	316
Recessive	12	7	rs505532	29084392	0.00005747	*CPVL*	80	122	61	95	284	160
Recessive	13	17	rs7219495	64722145	0.00006064	*PRKCA*	60	116	89	62	261	218
Recessive	14	13	rs17300865	95882524	0.00006164	*ABCC4*	1	97	167	31	160	350
Recessive	15	10	rs7076500	123021731	0.00006172		51	120	94	49	265	227
Recessive	16	12	rs10785056	74006401	0.00006194		16	137	112	85	247	209
Recessive	17	8	rs2976589	134334904	0.00006301		20	89	156	9	186	346

Recessive	18	2	rs876718	15808935	0.00006361		8	112	144	59	201	279
Recessive	19	2	rs10496907	142665064	0.00006443	*LRP1B*	6	130	129	52	207	281
Recessive	20	1	rs1832574	20149058	0.00006867		20	130	115	96	235	210

B/B, homozygote for the major allele for each SNP

Model, the genetic model in which candidate SNPs were selected by the GWAS; CHR, chromosome number; Position, chromosomal position (bp); Related gene, the nearest gene from the SNP site; A/A, homozygote for the minor allele for each SNP; A/B, heterozygote for each SNP; B/B, homozygote for the major allele for each SNP.

Table S3. Top 20 candidate SNPs selected from the GWAS for vomiting in all patients.

Model	Rank	CHR	SNP	Position	*p*	Related gene	Genotype (Nausea +)			Genotype (Nausea -)		
							A/A	A/B	B/B	A/A	A/B	B/B
Trend	1	2	rs13420654	79560656	0.000002095		0	29	120	1	43	613
Trend	2	18	exm1387565	50866195	0.000002217	*DCC*	1	28	120	1	45	611
Trend	3	5	rs4518389	124327862	0.000002404		16	77	56	163	346	146
Trend	4	5	rs1421691	164541466	0.00000336		6	51	92	9	127	520
Trend	5	2	exm185124	32641040	0.000004066	*BIRC6*	0	7	142	0	2	655
Trend	5	3	exm2047963	195974246	0.000004066	*PCYT1A*	0	7	142	0	2	655
Trend	5	12	rs1805772	9087081	0.000004066	*PHC1*	0	7	142	0	2	655
Trend	8	20	rs6013869	52619796	0.000009216	*BCAS1*	24	60	65	179	312	166
Trend	9	20	rs12624524	46557322	0.000009759		3	35	111	6	67	584
Trend	10	20	rs2299723	52622783	0.00001035	*BCAS1*	58	66	25	146	321	190
Trend	11	5	rs10440778	124329306	0.00001216		16	78	55	161	343	153
Trend	12	9	rs306772	124092355	0.00001335	*GSN*	3	46	100	4	110	543
Trend	13	2	rs2216322	56374910	0.00001457		11	58	80	21	167	469
Trend	14	20	rs6118234	8509458	0.00001568	*PLCB1*	29	66	54	40	303	314

Trend	15	20	rs7270072	57998583	0.00001769		25	88	36	68	304	285
Trend	16	2	rs1965757	166164887	0.00001807	*SCN2A*	53	74	22	142	329	186
Trend	17	4	rs28391069	167559106	0.00002093		1	9	139	0	8	649
Trend	18	15	rs10520799	96201031	0.00002426		0	15	134	0	17	640
Trend	19	19	rs751792	35157254	0.00002445		46	61	42	94	308	255
Trend	20	7	rs12704714	93930713	0.00002464		41	67	41	86	309	261

Dominant	1	5	rs10064687	109955062	0.00000906	*TMEM232*	28	62	59	171	343	141
Dominant	2	2	rs13420654	79560656	0.00000937		0	29	120	1	43	613
Dominant	3	20	rs7270072	57998583	0.00001178		25	88	36	68	304	285
Dominant	4	18	exm1387565	50866195	0.00001421	*DCC*	1	28	120	1	45	611
Dominant	5	20	rs6013869	52619796	0.00001427	*BCAS1*	24	60	65	179	312	166
Dominant	6	5	rs4317370	109910067	0.00001449	*TMEM232*	28	62	59	173	341	143
Dominant	7	5	rs1421691	164541466	0.00001715		6	51	92	9	127	520
Dominant	8	20	rs12624524	46557322	0.00001763		3	35	111	6	67	584
Dominant	9	2	rs12991861	55585915	0.0000206	*CCDC88A*	39	82	28	121	296	240
Dominant	10	12	rs4761419	76712538	0.00002599		17	81	50	45	264	348
Dominant	11	16	rs12595990	12471112	0.00002909	*SNX29*	22	56	71	131	333	193
Dominant	12	10	rs10748915	108058716	0.00003335		11	86	48	47	266	341
Dominant	13	9	rs13296679	71347988	0.00004011	*PIP5K1B*	17	86	46	78	254	323
Dominant	14	3	rs11710227	187753995	0.00004041		21	76	52	70	234	352
Dominant	15	3	exm2255887	187753889	0.00004112		21	76	52	70	235	352
Dominant	15	3	rs16862908	187753889	0.00004112		21	76	52	70	235	352
Dominant	17	19	rs11668036	35186128	0.00004865		13	48	87	81	312	263
Dominant	18	2	rs1045613	55516090	0.00005243	*CCDC88A*	39	80	30	120	292	245
Dominant	19	2	rs13019339	55589364	0.00005317	*CCDC88A*	48	78	23	149	301	207
Dominant	20	9	rs4743505	98827451	0.00005637		15	44	90	70	311	276

Recessive	1	20	rs6118234	8509458	0.000001949	*PLCB1*	29	66	54	40	303	314
Recessive	2	5	rs9292416	31021518	0.000006208		58	58	33	135	341	181
Recessive	3	19	rs751792	35157254	0.000006504		46	61	42	94	308	255
Recessive	4	9	rs7027092	95812707	0.00001309		55	59	33	129	324	194
Recessive	5	5	rs6869417	31020501	0.00001545		56	61	32	133	333	191
Recessive	6	19	rs1985840	54880076	0.00001764		18	49	82	19	247	391
Recessive	7	4	rs6448841	11861566	0.00001953		17	49	83	17	249	391
Recessive	7	4	rs12645979	11868097	0.00001953		17	50	82	17	251	389
Recessive	9	9	exm2266850	95812707	0.0000209		55	61	33	129	327	195
Recessive	10	3	rs9809465	16674026	0.00002504		25	55	69	37	308	312
Recessive	11	13	rs10508158	106247124	0.00003162		28	57	64	46	288	323
Recessive	12	19	rs8109293	21009270	0.00003279		57	55	37	140	354	162
Recessive	13	5	rs40654	9371411	0.00003672	*SEMA5A*	6	27	116	0	118	539
Recessive	14	17	rs7219495	64722145	0.00003791	*PRKCA*	40	61	48	82	316	259
Recessive	15	13	rs2028809	44523136	0.00004081		3	84	62	81	281	295
Recessive	16	20	rs2299723	52622783	0.00004292	*BCAS1*	58	66	25	146	321	190
Recessive	17	1	rs12045323	11764281	0.00004639	*C1orf187*	9	69	71	124	307	226
Recessive	18	11	rs1025139	122146533	0.00004693		20	44	85	26	247	384
Recessive	19	7	rs12704714	93930713	0.00005179		41	67	41	86	309	261
Recessive	20	11	rs10830263	89100329	0.00005396	*NOX4*	6	74	69	104	292	261

B/B, homozygote for the major allele for each SNP

Model, the genetic model in which candidate SNPs were selected by the GWAS; CHR, chromosome number; Position, chromosomal position (bp); Related gene, the nearest gene from the SNP site; A/A, homozygote for the minor allele for each SNP; A/B, heterozygote for each SNP; B/B, homozygote for the major allele for each SNP.

Table S4. Top 20 candidate SNPs selected from the GWAS for PONV in all patients.

Model	Rank	CHR	SNP	Position	*p*	Related gene	Genotype (Nausea +)			Genotype (Nausea -)		
							A/A	A/B	B/B	A/A	A/B	B/B
Trend	1	20	rs6021693	50694287	0.000002062		48	156	76	53	245	228
Trend	2	10	rs7097450	123878029	0.000007302	*TACC2*	3	16	260	0	6	518
Trend	3	13	rs645833	48724256	0.000007539		0	31	249	0	17	509
Trend	4	16	rs9925934	17028247	0.00001144		1	67	212	3	58	465
Trend	5	10	rs7073770	15417537	0.00001479		1	13	266	1	83	442
Trend	6	20	rs743093	50695849	0.00001576		35	146	95	34	235	254
Trend	7	8	rs17296667	125296872	0.00001752		2	90	188	9	82	435
Trend	8	17	rs1978136	32377698	0.00001915	*ACCN1*	60	144	76	65	252	209
Trend	9	16	rs6498643	17050874	0.0000212		2	73	205	5	66	454
Trend	10	17	rs886359	32399974	0.00002162	*ACCN1*	38	141	101	128	267	131
Trend	11	20	exm-rs6013382	50702633	0.00002242	*ZFP64*	35	148	97	35	235	256
Trend	11	20	rs6013382	50702633	0.00002242	*ZFP64*	35	148	97	35	235	256
Trend	13	21	rs2836505	39918553	0.00002353	*ERG*	17	101	162	56	248	222
Trend	14	1	rs11582464	30745268	0.00002553		8	87	185	5	105	416
Trend	15	17	rs12602886	32392346	0.00002597	*ACCN1*	38	141	101	128	266	132
Trend	16	18	rs1944967	39531139	0.00002609		61	136	82	70	231	225
Trend	17	20	rs2273382	50705211	0.00002661	*ZFP64*	35	148	97	35	236	255
Trend	18	10	rs7911912	15439080	0.00002989		1	41	238	7	140	378

Trend	19	5	rs7719723	2137758	0.00003019		30	134	116	119	242	165
Trend	20	5	rs28558979	68820348	0.00003064	*OCLN*	13	92	175	54	218	254
Dominant	1	8	rs17296667	125296872	0.0000009951		2	90	188	9	82	435
Dominant	2	10	rs7073770	15417537	0.000002425		1	13	266	1	83	442
Dominant	3	16	rs9925934	17028247	0.000004867		1	67	212	3	58	465
Dominant	4	16	rs6498643	17050874	0.000005539		2	73	205	5	66	454
Dominant	5	20	rs6021693	50694287	0.000006286		48	156	76	53	245	228
Dominant	6	6	rs600613	98206805	0.000009947		52	157	71	77	233	216
Dominant	7	4	rs4689343	6136653	0.00001392	*JAKMIP1*	0	3	277	0	41	485
Dominant	8	13	rs645833	48724256	0.00001557		0	31	249	0	17	509
Dominant	9	9	rs7869201	5551261	0.00001585	*PDCD1LG2*	40	142	98	61	197	268
Dominant	10	3	exm333077	98250986	0.00001886	*GPR15*	31	101	148	67	264	194
Dominant	11	3	rs2230344	98250986	0.00001936	*GPR15*	31	101	148	67	264	195
Dominant	12	7	rs17156585	28366100	0.00001976	*CREB5*	4	84	192	7	87	431
Dominant	13	1	rs10900553	204108163	0.00002033	*ETNK2*	33	127	120	86	294	146
Dominant	14	7	rs6978238	28370058	0.00002244	*CREB5*	2	83	195	7	83	436
Dominant	15	10	rs17102809	123728523	0.00002278	*NSMCE4A*	20	140	120	42	175	308
Dominant	16	1	rs2970580	212097521	0.00002317		5	43	232	12	150	364
Dominant	17	17	rs7212872	55274630	0.00002401		13	119	148	20	147	359
Dominant	18	21	rs2836505	39918553	0.00002409	*ERG*	17	101	162	56	248	222

Dominant	19	4	rs2171726	11922712	0.00002566		10	68	201	24	202	299
Dominant	20	10	rs7911912	15439080	0.00002612		1	41	238	7	140	378
Recessive	1	6	rs9403523	144004981	0.000005161	*PHACTR2*	15	146	119	84	234	208
Recessive	2	6	rs9403521	143994199	0.000006421	*PHACTR2*	13	145	122	78	234	214
Recessive	3	22	rs4823376	45171729	0.00001186	*PRR5-ARHGAP8,ARHGAP8*	11	125	144	70	183	273
Recessive	4	17	rs7219495	64722145	0.000012980	*PRKCA*	64	126	90	58	251	217
Recessive	5	11	rs10831744	12189013	0.00001801	*MICAL2*	15	148	117	80	238	208
Recessive	6	7	rs505532	29084392	0.00002162	*CPVL*	85	129	64	90	277	157
Recessive	7	7	rs4141275	29079870	0.00002244	*CPVL*	85	132	63	90	281	155
Recessive	8	5	rs7719723	2137758	0.00002445		30	134	116	119	242	165
Recessive	9	13	rs17300865	95882524	0.00002983	*ABCC4*	1	100	179	31	157	338
Recessive	10	12	rs10785056	74006401	0.00003015		17	141	122	84	243	199
Recessive	11	6	rs9322497	155382807	0.00003393		52	108	120	44	253	229
Recessive	12	20	rs6122536	45543676	0.000035050	*EYA2*	30	136	108	119	234	169
Recessive	13	12	exm1052074	132325239	0.000038730	*MMP17*	78	123	79	81	288	157
Recessive	14	12	rs6598163	132325239	0.000039170	*MMP17*	78	123	79	81	287	157
Recessive	15	9	rs2270202	131191403	0.00003921	*CERCAM*	26	156	98	107	256	163
Recessive	16	8	rs7816614	5344373	0.00004251		19	75	185	6	156	358
Recessive	17	19	rs1985840	54880076	0.00005274		25	95	160	12	201	313
Recessive	18	4	rs7672861	142165741	0.00006145		32	106	142	20	209	296

Recessive	19	4	rs12650715	185776850	0.00007079		21	127	132	92	203	231
Recessive	20	17	rs8067877	64731178	0.000071880	*PRKCA*	62	126	92	59	249	218
Recessive	20	17	rs6504452	64731402	0.000071880	*PRKCA*	62	126	92	59	249	218

B/B, homozygote for the major allele for each SNP

Model, the genetic model in which candidate SNPs were selected by the GWAS; CHR, chromosome number; Position, chromosomal position (bp); Related gene, the nearest gene from the SNP site; A/A, homozygote for the minor allele for each SNP; A/B, heterozygote for each SNP; B/B, homozygote for the major allele for each SNP.

Table S5. Top 20 candidate SNPs selected from the GWAS for nausea in patients who received propofol.

Model	Rank	CHR	SNP	Position	*p*	Related gene	Genotype (Nausea +)			Genotype (Nausea -)		
							A/A	A/B	B/B	A/A	A/B	B/B
Trend	1	17	rs11079308	55604585	0.000001544	*MSI2*	9	54	86	1	67	225
Trend	2	9	rs4977887	23718520	0.000001599	*ELAVL2*	10	57	82	50	148	95
Trend	3	6	rs7356878	40494855	0.000001969	*LRFN2*	52	77	20	56	145	92
Trend	4	6	rs1433742	40503796	0.000003573	*LRFN2*	26	75	48	23	112	158
Trend	5	5	rs7719723	2137758	0.000004131		11	71	67	73	134	86
Trend	6	6	rs382023	40491240	0.000005878	*LRFN2*	15	76	58	75	151	67
Trend	7	15	rs11072390	73136716	0.000006207		42	72	35	39	134	120
Trend	8	7	rs1982436	134470632	0.000007065	*CALD1*	5	63	81	5	65	223
Trend	9	6	rs403606	40493903	0.000007295	*LRFN2*	15	73	61	76	144	73
Trend	10	10	rs10901376	127085818	0.00001249		0	9	140	4	62	227
Trend	11	17	rs11655926	35243370	0.00001366		15	68	66	11	93	189
Trend	12	6	rs909988	40489573	0.00001982	*LRFN2*	35	85	29	35	151	107

Trend	13	15	rs12439063	73150166	0.00002141		22	80	47	24	113	156
Trend	14	23	rs7879223	95721436	0.00002616		0	12	126	0	1	234
Trend	15	6	rs2235705	40486159	0.00002706	*LRFN2*	25	83	41	19	145	129
Trend	16	18	rs540291	77585900	0.00002834		31	78	40	35	119	139
Trend	17	3	exm-rs2286720	42448471	0.00003213	*LYZL4*	3	58	88	27	147	119
Trend	17	3	rs2286720	42448471	0.00003213	*LYZL4*	3	58	88	27	147	119
Trend	19	10	rs10828239	21679527	0.00003652		31	86	32	38	130	123
Trend	20	3	rs1529047	98252027	0.00003978		16	63	70	11	91	191

Dominant	1	7	rs1982436	134470632	0.00000462	*CALD1*	5	63	81	5	65	223
Dominant	2	9	rs4977887	23718520	0.000005809	*ELAVL2*	10	57	82	50	148	95
Dominant	3	10	rs10901376	127085818	0.000006565		0	9	140	4	62	227
Dominant	4	9	rs4143813	5534243	0.000006694	*PDCD1LG2*	9	63	77	14	63	216
Dominant	5	18	rs1942295	66544809	0.00000876	*CCDC102B*	10	39	100	21	141	131
Dominant	6	10	rs10828239	21679527	0.0000139		31	86	32	38	130	123
Dominant	7	15	rs12439063	73150166	0.00001412		22	80	47	24	113	156
Dominant	8	6	rs1433742	40503796	0.00001442	*LRFN2*	26	75	48	23	112	158
Dominant	9	5	rs11749532	55002265	0.00001672	*SLC38A9*	28	87	34	46	119	128
Dominant	10	6	rs7356878	40494855	0.0000279	*LRFN2*	52	77	20	56	145	92
Dominant	11	8	rs12550470	55344415	0.00002795		15	58	76	43	162	88
Dominant	12	11	rs7933966	32875597	0.00002988	*PRRG4*	30	58	61	64	166	63
Dominant	12	11	exm-rs10767971	32895664	0.00002988		30	58	61	64	166	63

Dominant	12	11	rs10767971	32895664	0.00002988		30	58	61	64	166	63
Dominant	15	8	rs13258851	55330814	0.00003278		11	48	90	30	148	115
Dominant	16	10	rs511991	29449129	0.00003284		24	67	58	58	177	58
Dominant	17	1	rs6658239	240511965	0.00003429	*FMN2*	15	82	52	27	102	164
Dominant	18	18	rs540291	77585900	0.00003786		31	78	40	35	119	139
Dominant	19	10	rs2781256	25616473	0.000038	*GPR158*	5	62	82	8	66	219
Dominant	20	8	rs10099341	55368646	0.00003836		17	59	73	46	162	84

Recessive	1	5	rs7719723	2137758	0.000005031		11	71	67	73	134	86
Recessive	2	6	rs9403521	143994199	0.00001184	*PHACTR2*	4	78	67	46	121	126
Recessive	3	7	rs4141275	29079870	0.00001193	*CPVL*	52	68	29	47	161	85
Recessive	4	13	rs1782805	40946993	0.0000191	*TTL*	16	48	85	4	97	192
Recessive	5	8	rs2942213	23427919	0.00001952	*SLC25A37*	4	74	71	45	120	128
Recessive	6	6	rs9403523	144004981	0.00002179	*PHACTR2*	5	80	64	48	121	124
Recessive	7	3	rs6782799	119610793	0.00003118	*GSK3B*	16	86	47	81	127	85
Recessive	7	3	rs17204878	119776880	0.00003118	*GSK3B*	16	86	47	81	127	85
Recessive	9	4	rs9306994	76576116	0.00003415	*G3BP2*	0	63	86	25	98	169
Recessive	10	5	rs4362905	52927597	0.00003802	*NDUFS4*	2	71	76	35	115	143
Recessive	10	5	rs11745611	53011223	0.00003802		2	73	74	35	116	142
Recessive	12	12	rs10437774	20139160	0.00004546		12	77	59	69	136	88
Recessive	13	9	rs10816556	110451579	0.0000477		9	34	106	0	74	219
Recessive	13	9	rs10759254	110451637	0.0000477		9	34	106	0	74	219

Recessive	15	3	rs7651125	6401420	0.0000482		3	65	81	39	105	148
Recessive	16	11	rs482795	116227687	0.00004855		14	84	51	74	130	89
Recessive	17	3	rs6438552	119631814	0.00004886	*GSK3B*	16	85	47	80	128	85
Recessive	18	21	rs2268284	36220679	0.00006101	*RUNX1*	48	69	32	44	169	79
Recessive	19	6	rs403606	40493903	0.00006134	*LRFN2*	15	73	61	76	144	73
Recessive	20	8	rs837065	130853447	0.0000622	*FAM49B*	10	86	53	62	139	92

B/B, homozygote for the major allele infor each SNP

Model, the genetic model in which candidate SNPs were selected by the GWAS; CHR, chromosome number; Position, chromosomal position (bp); Related gene, the nearest gene from the SNP site; A/A, homozygote for the minor allele for each SNP; A/B, heterozygote for each SNP; B/B, homozygote for the major allele for each SNP.

Table S6. Top 20 candidate SNPs selected from the GWAS for PONV in patients who received propofol.

Model	Rank	CHR	SNP	Position	*p*	Related gene	Genotype (Nausea +)			Genotype (Nausea -)		
							A/A	A/B	B/B	A/A	A/B	B/B
Trend	1	13	rs1752136	48726219	0.000002425		0	25	135	0	9	273
Trend	2	6	rs7356878	40494855	0.000002695	*LRFN2*	55	82	23	53	140	89
Trend	3	17	rs11079308	55604585	0.000003682	*MSI2*	9	57	94	1	64	217
Trend	4	9	rs4977887	23718520	0.000004479	*ELAVL2*	11	64	85	49	141	92
Trend	5	15	rs11072390	73136716	0.000005056		44	78	38	37	128	117
Trend	6	2	rs1922717	11024358	0.00000913		4	41	115	0	35	247
Trend	7	5	rs7719723	2137758	0.000009416		14	75	71	70	130	82
Trend	8	6	rs403606	40493903	0.00001074	*LRFN2*	17	79	64	74	138	70
Trend	9	6	rs1433742	40503796	0.00001093	*LRFN2*	28	77	55	21	110	151
Trend	10	3	exm-rs2286720	42448471	0.00001378	*LYZL4*	3	63	94	27	142	113
Trend	10	3	rs2286720	42448471	0.00001378	*LYZL4*	3	63	94	27	142	113

Trend	12	7	rs4141275	29079870	0.00001401	*CPVL*	55	75	30	44	154	84
Trend	13	3	rs1529047	98252027	0.00001439		16	70	74	11	84	187
Trend	14	6	rs382023	40491240	0.00001476	*LRFN2*	17	83	60	73	144	65
Trend	15	15	rs12439063	73150166	0.00001589		23	86	51	23	107	152
Trend	16	5	rs28558979	68820348	0.00001621	*OCLN*	3	52	105	25	126	131
Trend	17	17	rs4644888	64745690	0.00002141	*PRKCA*	22	64	74	65	145	72
Trend	18	17	rs8070561	64747056	0.00002539	*PRKCA*	22	64	74	64	146	72
Trend	19	3	rs4682737	42484782	0.0000284		3	64	93	27	141	114
Trend	20	2	rs17036833	69851974	0.00002911	*AAK1*	1	34	125	0	23	259
Dominant	1	18	rs1942295	66544809	0.000004643	*CCDC102B*	10	43	107	21	137	124
Dominant	2	13	rs1752136	48726219	0.000007011		0	25	135	0	9	273
Dominant	3	15	rs12439063	73150166	0.000007709		23	86	51	23	107	152
Dominant	4	1	rs1571549	86050965	0.000011		25	68	67	67	154	61
Dominant	5	17	rs4644888	64745690	0.00001418	*PRKCA*	22	64	74	65	145	72
Dominant	5	17	rs8070561	64747056	0.00001418	*PRKCA*	22	64	74	64	146	72
Dominant	7	14	rs4983168	26851322	0.00002622		28	93	39	46	111	125
Dominant	8	7	rs1982436	134470632	0.00002819	*CALD1*	5	65	90	5	63	214
Dominant	9	23	rs1318834	39345412	0.00002928		43	83	20	48	104	75
Dominant	10	1	rs6658239	240511965	0.0000307	*FMN2*	17	86	57	25	98	159
Dominant	11	9	rs4977887	23718520	0.00003229	*ELAVL2*	11	64	85	49	141	92
Dominant	12	4	rs2380737	37536119	0.00003232	*C4orf19*	0	1	159	2	26	254
Dominant	13	3	rs9837095	167323709	0.00003708	*WDR49*	2	27	131	14	89	178
Dominant	14	3	exm363153	167320010	0.0000384	*WDR49*	2	27	131	14	89	179
Dominant	15	16	rs2074365	2964283	0.00004003	*FLYWCH1*	18	89	51	36	97	149

Dominant	16	4	rs11946898	181926518	0.00004213		34	96	30	54	123	105
Dominant	17	9	rs7869201	5551261	0.00004234	*PDCD1LG2*	26	84	50	36	101	145
Dominant	18	8	rs13258851	55330814	0.00004506		12	53	95	29	143	110
Dominant	19	10	rs10828239	21679527	0.00005148		31	92	37	38	124	118
Dominant	20	3	rs1529047	98252027	0.00005312		16	70	74	11	84	187
Recessive	1	8	rs2942213	23427919	0.000005171	*SLC25A37*	4	81	75	45	113	124
Recessive	2	7	rs4141275	29079870	0.000009253	*CPVL,CPVL*	55	75	30	44	154	84
Recessive	3	5	rs4362905	52927597	0.0000095	*NDUFS4*	2	78	80	35	108	139
Recessive	3	5	rs11745611	53011223	0.0000095		2	80	78	35	109	138
Recessive	5	7	rs12698219	158145606	0.00001116	*PTPRN2*	11	32	117	0	51	231
Recessive	6	3	rs7651125	6401420	0.00001256		3	69	88	39	101	141
Recessive	7	5	rs567	52979097	0.00001627	*NDUFS4*	2	78	80	34	109	139
Recessive	8	1	rs1195866	81678254	0.00001682		16	47	96	3	98	178
Recessive	9	17	rs7219495	64722145	0.00002003	*PRKCA*	41	75	44	28	141	113
Recessive	10	11	rs482795	116227687	0.00002028		15	93	52	73	121	88
Recessive	11	5	rs7719723	2137758	0.00002501		14	75	71	70	130	82
Recessive	12	3	rs6796561	6409144	0.00002696		0	57	103	23	99	160
Recessive	12	10	rs2420953	123483879	0.00002696		0	65	95	23	102	157
Recessive	12	10	rs2935689	123486626	0.00002696		0	65	95	23	102	157
Recessive	15	7	rs505532	29084392	0.00003413	*CPVL,CPVL*	55	73	31	47	149	84
Recessive	16	8	rs2942201	23419176	0.0000366	*SLC25A37*	3	76	81	36	105	141
Recessive	17	1	rs11165660	96993561	0.00003954		16	90	54	73	127	80
Recessive	18	14	rs10483614	53151428	0.00004454	*ERO1L*	20	62	78	7	114	161
Recessive	19	7	rs6964887	17728255	0.00004826		50	69	38	42	147	92
Recessive	20	5	rs6886811	53020039	0.00005066		2	75	82	32	106	144

Model, the genetic model in which candidate SNPs were selected by the GWAS; CHR, chromosome number; Position, chromosomal position (bp); Related gene, the nearest gene from the SNP site; A/A, homozygote for the minor allele for each SNP; A/B, heterozygote for each SNP; B/B, homozygote for the major allele for each SNP.

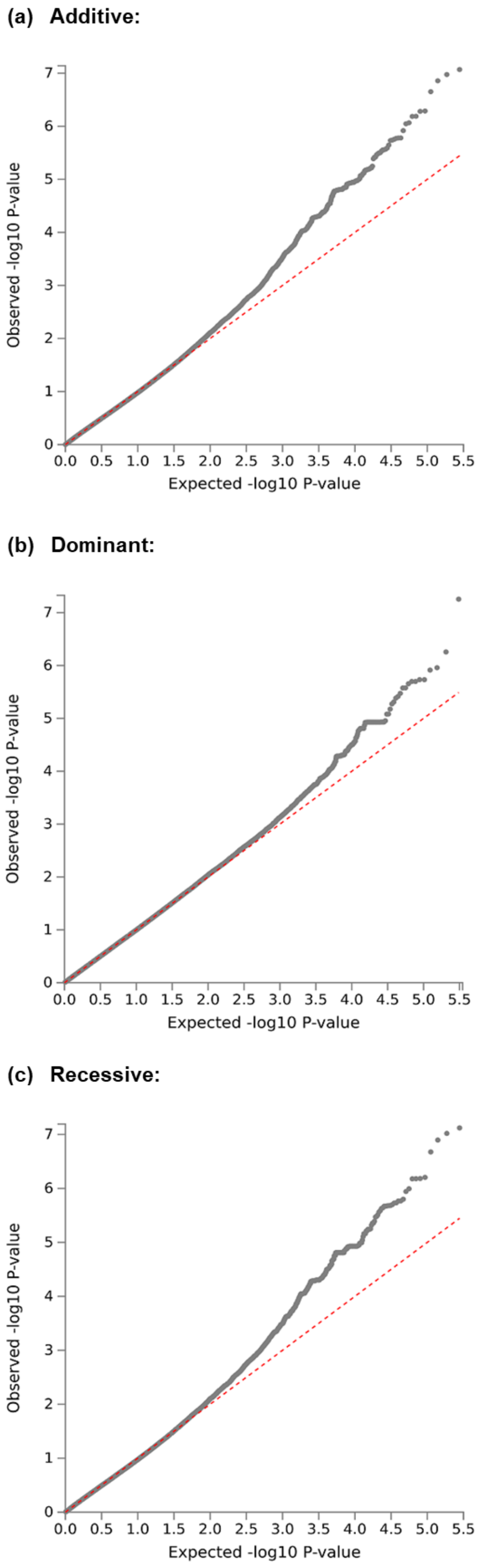

Figure S1. Log quantile-quantile (QQ) *p*-value plot as a result of the GWAS for the frequency of nausea in all patients. (a) Plot of the results from the additive model. (b) Plot of the results from the dominant model. (c) Plot of the results from the recessive model.

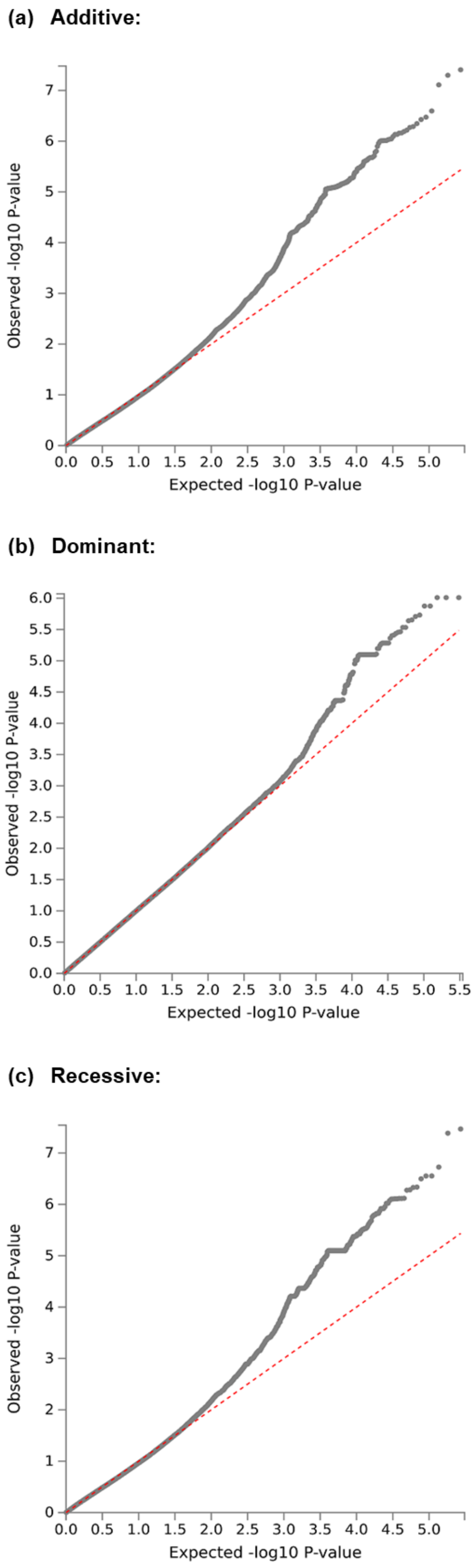

Figure S2. Log quantile-quantile (QQ) *p*-value plot as a result of the GWAS for the frequency of nausea in patients with propofol. (a) Plot of the results from the additive model. (b) Plot of the results from the dominant model. (c) Plot of the results from the recessive model.

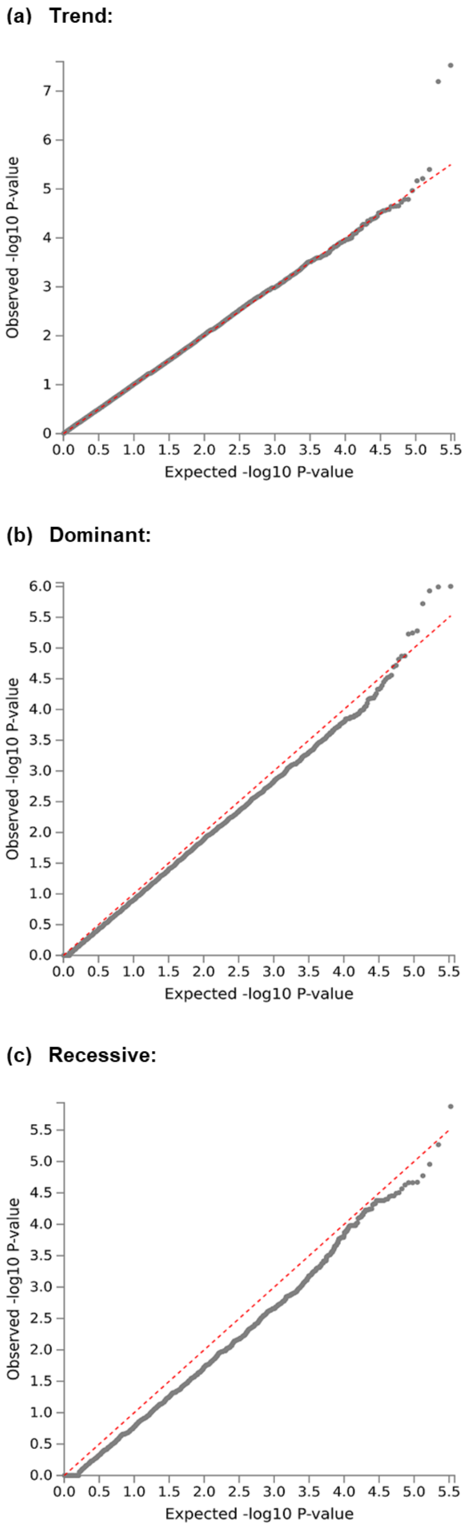

Figure S3. Log quantile-quantile (QQ) *p*-value plot as a result of the GWAS for vomiting in patients who received propofol. (a) Plot of the results from the trend model. (b) Plot of the results from the dominant model. (c) Plot of the results from the recessive model.

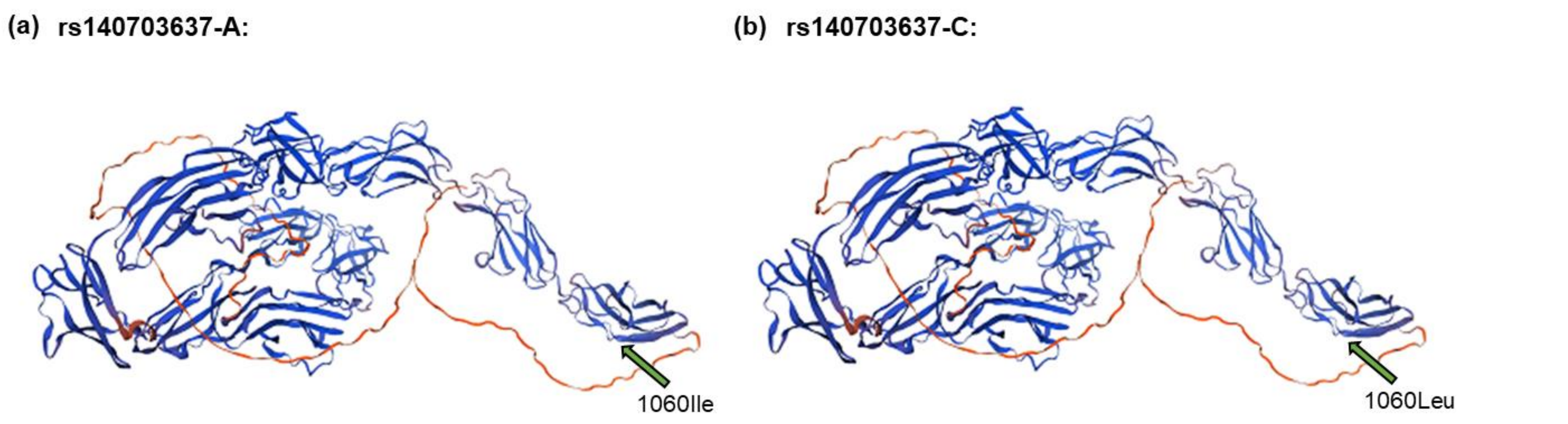

Figure S4. Protein structures of contactin 5 predicted from amino acid sequence (NCBI accession no. NP_001230199.1). (a) Protein structure predicted from amino acid sequence, including isoleucine residue, that corresponds to the A allele of the rs140703637 SNP. (b) Protein structure predicted from amino acid sequence, including leucine residue, that corresponds to the C allele of the rs140703637 SNP. The amino acids that correspond to the position of the rs140703637 SNP are indicated by the arrowheads.

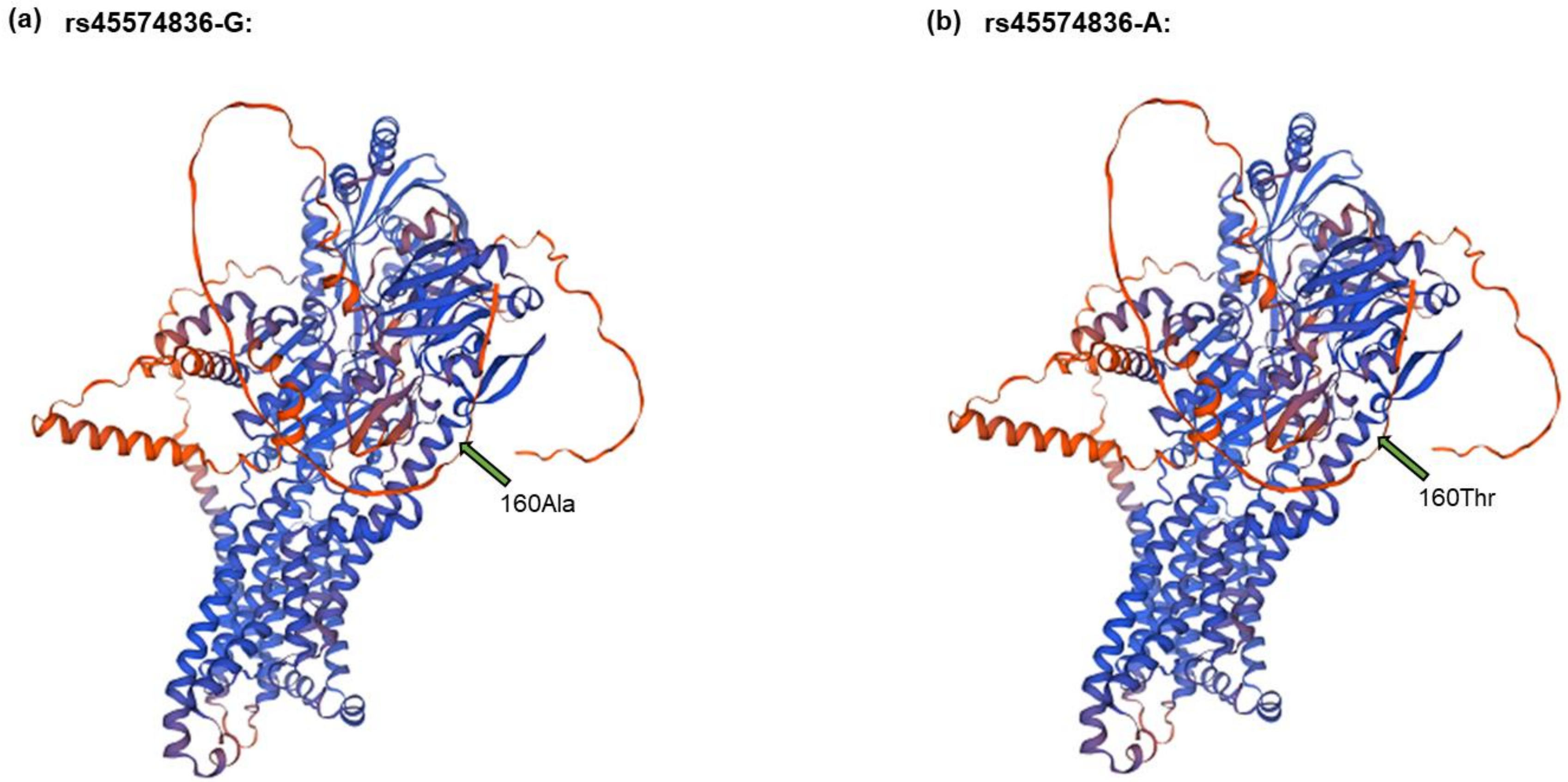

Figure S5. Protein structures of ATPase phospholipid transporting 8B3 predicted from amino acid sequence (NCBI accession no. NP_001171473.1). (a) Protein structure predicted from amino acid sequence, including alanine residue, that corresponds to the G allele of the rs45574836 SNP. (b) Protein structure predicted from amino acid sequence, including threonine residue, that corresponds to the A allele of the rs45574836 SNP. The amino acids that correspond to the position of the rs140703637 SNP are indicated by the arrowheads.
